# Alterations of Cardiac Protein Kinases in Cyclic Nucleotide-Dependent Signaling Pathways in Human Ischemic Heart Failure

**DOI:** 10.3389/fcvm.2022.919355

**Published:** 2022-06-17

**Authors:** Chunguang Wang, Juuso H. Taskinen, Heli Segersvärd, Katariina Immonen, Riikka Kosonen, Johanna M. Tolva, Mikko I. Mäyränpää, Petri T. Kovanen, Vesa M. Olkkonen, Juha Sinisalo, Mika Laine, Ilkka Tikkanen, Päivi Lakkisto

**Affiliations:** ^1^Minerva Foundation Institute for Medical Research, Biomedicum Helsinki 2 U, Helsinki, Finland; ^2^Transplantation Laboratory, Department of Pathology, University of Helsinki, Helsinki, Finland; ^3^Department of Pathology, University of Helsinki and Helsinki University Hospital, Helsinki, Finland; ^4^Atherosclerosis Research Laboratory, Wihuri Research Institute, Helsinki, Finland; ^5^Department of Anatomy, Faculty of Medicine, University of Helsinki, Helsinki, Finland; ^6^Heart and Lung Center, University of Helsinki and Helsinki University Hospital, Helsinki, Finland; ^7^Abdominal Center, Nephrology, University of Helsinki and Helsinki University Hospital, Helsinki, Finland; ^8^Clinical Chemistry and Hematology, University of Helsinki and Helsinki University Hospital, Helsinki, Finland

**Keywords:** ischemic heart disease, ischemic heart failure, cardiac kinome, cAMP-dependent protein kinase, cGMP-dependent protein kinase, second messenger intracellular signaling, natriuretic peptide

## Abstract

**Objectives:**

Impaired protein kinase signaling is a hallmark of ischemic heart disease (IHD). Inadequate understanding of the pathological mechanisms limits the development of therapeutic approaches. We aimed to identify the key cardiac kinases and signaling pathways in patients with IHD with an effort to discover potential therapeutic strategies.

**Methods:**

Cardiac kinase activity in IHD left ventricle (LV) and the related signaling pathways were investigated by kinomics, transcriptomics, proteomics, and integrated multi-omics approach.

**Results:**

Protein kinase A (PKA) and protein kinase G (PKG) ranked on top in the activity shift among the cardiac kinases. In the IHD LVs, PKA activity decreased markedly compared with that of controls (62% reduction, *p* = 0.0034), whereas PKG activity remained stable, although the amount of PKG protein increased remarkably (65%, *p* = 0.003). mRNA levels of adenylate cyclases (*ADCY 1, 3, 5, 9*) and cAMP-hydrolysing phosphodiesterases (*PDE4A, PDE4D*) decreased significantly, although no statistically significant alterations were observed in that of PKGs (*PRKG1* and *PRKG2*) and guanylate cyclases (*GUCYs*). The gene expression of natriuretic peptide CNP decreased remarkably, whereas those of BNP, ANP, and neprilysin increased significantly in the IHD LVs. Proteomics analysis revealed a significant reduction in protein levels of “Energy metabolism” and “Muscle contraction” in the patients. Multi-omics integration highlighted intracellular signaling by second messengers as the top enriched Reactome pathway.

**Conclusion:**

The deficiency in cAMP/PKA signaling pathway is strongly implicated in the pathogenesis of IHD. Natriuretic peptide CNP could be a potential therapeutic target for the modulation of cGMP/PKG signaling.

## Introduction

Ischemic heart failure (IHF), the clinical end point of ischemic heart disease (IHD), remains the leading cause of death globally. A universal definition of heart failure (HF) has been proposed as a clinical syndrome with symptoms and/or signs caused by a structural and/or functional cardiac abnormality and corroborated by elevated natriuretic peptide levels and/or objective evidence of pulmonary or systemic congestion (1). Despite its growing prevalence and increasing needs to develop personalized therapeutic approaches, progress is limited to symptomatic management due to fragmented research and inadequate understanding of the pathological mechanisms. Identification and characterization of key underpinnings in the pathogenesis of IHD with an effort to find potential therapeutic strategies is of great importance.

Protein kinases play crucial roles in heart functions, including contraction, metabolism, ion fluxes, and gene transcription. Impaired or insufficient protein kinase signaling due to alterations in activity, expression, and compartmentalization is considered as the hallmark of most cardiac diseases, including the IHF (2). The mammalian kinome possesses over 500 highly conserved kinases being classified into eight superfamilies (AGC, Atypical, CAMK, CK1, CMGC, STE, TK, TKL) according to homologies within their catalytic domains (3). Yet, the studies of the cardiac kinome and its role in cardiac pathophysiology have been very limited, especially the systematic investigations in human ischemic failing hearts (3, 4). Fuller et al. described the kinome in rat cardiomyocytes and the altered kinase expression in failing human hearts (3). An integrated transcriptome and bioinformatics approach has been shown to identify novel cardiac kinases, which may play a role in heart failure using mouse hearts (4). Integrated approaches can aid in revealing the underlying mechanisms at multiple omics levels and thus yield better understanding of the complex human diseases (5–7). In an effort to identify novel cardiac kinases potentially involved in IHD and to expand our understanding of the cardiac kinome to search for potential therapeutic targets, we compared control and end-stage IHD human hearts by using an integrated kinomics, transcriptomics, and proteomics approach, which allowed evaluation of the global profile changes.

## Materials and Methods

### Human Left Ventricular Samples

The cardiac left ventricular (LV) tissues were obtained from the patients with end-stage IHD (IHD n=8) who underwent cardiac transplantation in the Helsinki University Hospital. All samples were collected transmyocardially in the non-infarct area from the LV free wall between the left anterior descending artery (LAD) and the left circumflex artery (LCX). The LV samples were snap frozen in isopentane (2-methylbutane, pre-cooled in liquid nitrogen), and kept in liquid nitrogen until being transferred to−80°C for further use. The investigation conformed to the Declaration of Helsinki. The Ethics Committee of Helsinki and Uusimaa Hospital District approved the study. The cardiac samples were collected during 2014–2019, and a written informed consent was obtained from each patient. Control samples (Ctrl *n* = 8) were derived from the LVs of organ donors without cardiac disease, whose hearts could not be used as whole organ grafts due to tissue type or size mismatch. The National Authority for Medicolegal Affairs approved the usage of tissues from organ donors.

### PamChip Kinase Activity Profiling

#### Preparation of Protein Samples

Snap-frozen LV tissues (IHD *n* = 8, Ctrl *n* = 8) were homogenized in liquid nitrogen using mortar and pestle. The pulverized samples were collected and left on ice for 2 min before adding 100 μl of cold M-PER Mammalian Extraction Buffer (Thermo Fisher Scientific) containing Halt phosphatase inhibitor (1:100) and Halt protease inhibitor (1:100). Lysates were incubated for 30 min on ice and pipetted up and down every 10 min to promote lysis. Samples were centrifuged for 15 min at 16,000 × *g* at +4°C. Supernatants were collected, divided into aliquots, and stored at −80°C. Protein concentrations were determined using Pierce BCA Protein Assay Kit (Thermo Fisher Scientific).

#### Protein Kinase Activity Profiling

Kinase activity profiling was done on a PamStation12 System (PamGene International BV) using standard protocols for serine threonine kinase (STK) and protein tyrosine kinase (PTK) assays (Serine Threonine Kinase PamChip with STK Reagent Kit and Protein Tyrosine Kinase PamChip with PTK Reagent Kit). For each STK assay, 1 μg of protein was added in the reaction mixture. For each PTK assay, 7.5 μg of protein was present. To prevent non-specific binding, PamChip array was blocked with 2% bovine serum albumin.

#### Signal Quantification

Fluorescence signal intensities for all peptides were analyzed using BioNavigator® software (PamGene International BV). Quality controls were performed to exclude defective arrays. A differential analysis for each IHD vs. Ctrl pair was carried out using the BioNavigator® and R software. Combat-corrected data were used for statistical analysis. A two-Group (unpaired) *T*-test (2G) tool was used to generate a list of differentially phosphorylated peptides. The tool investigated the size and significance of the effect, by using an (unpaired) *T-*test between the IHD and Ctrl groups.

#### Upstream Kinase and Pathway Analysis

BioNavigator® software was used to perform upstream STK and PTK analysis by comparing differentially phosphorylated peptides between the IHD and Ctrl groups. Upstream Kinase Analysis tool (PamGene International BV, in-house method) was used to generate a ranking list of putative kinases responsible for the differences in peptide phosphorylation. Peptides being significantly and differently phosphorylated between the IHD and Ctrl groups were used for possible canonical pathways and networks study with Clarivate Analytics® GeneGO Pathway analysis tool. The top 10 significant pathways were identified and relevant signaling networks were assembled based on manually curated objects generated by log fold-change data. Pathways were ranked by -log (p). Significant effects (*p* < 0.05) were identified by fitting a model for the conditions, which performs a Dunnet's test for multiple conditions against a single control.

### Western Blotting and PKA/PKG Activity Measurements

#### Western Blotting

Protein kinase expression and phosphorylation were validated by Western blotting (IHD *n* = 7, Ctrl *n* = 7). Human heart LVs were homogenized with OMNI Bead Ruptor (Omni International) in extraction buffer (10 mM Tris-HCl, pH 7.4, containing 100 mM NaCl, 10 mM KCl, 8 mM Na_2_HPO_4_, 3 mM MgCl_2_, 1% NP-40) with 1 × Halt Phosphatase Inhibitor and 1 × Halt Protease Inhibitor (Thermo Scientific). Lysates (20 μg of protein per lane) were resolved by sodium dodecyl sulfate–polyacrylamide gel electrophoresis under reducing conditions and transferred onto polyvinylidene difluoride membranes (Bio-Rad). Membrane blocking was performed with 5% bovine serum albumin in TBST (1 × Tris-buffered saline, 0.1% Tween® 20). After primary antibody incubation (Supplementary Table 1), protein detection was performed with anti-mouse or anti-rabbit HRP-conjugated secondary antibodies (1:2,000, Jacksom ImmunoResearch Laboratories, Inc.). The signals were quantified using the ChemiDoc Imager (Bio-Rad) and normalized with total protein amount loaded in each lane. *T* tests (and non-parametric tests) in the Prism software were used to compare the variables in different groups. *P*-values < 0.05 were considered significant.

#### PKA and PKG Activity Measurements

The PKA activity was measured (IHD *n* = 7, Ctrl *n* = 6) using PKA Colorimetric Activity Kit (ThermoFisher Scientific) according to the manufacturer's instruction. Conditions were optimized to ensure that the reaction rate was linear at a proper dilution. The PKG activity was measured (IHD *n* = 7, Ctrl *n* = 7) using CycLex Cyclic GMP dependent protein kinase (cGK) Assay Kit (MBL). Mann–Whitney U test was used to compare the variables in different groups. *P*-values < 0.05 were considered significant.

### RNA Sequencing Analysis

To extract RNA, about 50 mg of snap-frozen LV (IHD *n* = 8, Ctrl *n* = 7) were cut into pieces on dry ice and homogenized with OMNI Bead Ruptor for 30 s (6 m/s) in 0.7 ml of QIAzol reagent (Qiagen). After removing debris with centrifugation, the lysate was transferred to a fresh tube and proceeded to RNA isolation using the miRNeasy Mini Kit (Qiagen). To ensure quality, extracted RNA was analyzed by TapeStation 4200 (Agilent Technologies, Inc.) at Biomedicum Functional Genomics Unit (FuGU, HiLIFE), University of Helsinki. All RNA integrity numbers (RINs) were ≥ 7.6. High-throughput mRNA-sequencing was performed at FuGU using Illumina NextSeq High Output 1 x 75 bp single-end reads (two flow cells), providing up to 40–50 M reads per sample. The data quality was checked by FastQC (http://www.bioinformatics.babraham.ac.uk/projects/fastqc/) and summarized with MultiQC (8). A light quality trimming was applied to the data with the Trimmomatic software (9). The sample reads were aligned against the reference genome GRCh38.p13 (Genome reference consortium human build 38 patch release 13, https://www.gencodegenes.org/human/release_33.html) with STAR-aligner (10). Alignment statistics was collected with the Qualimap tool (11) and read quantifications were calculated by using featureCounts (12). The differential expression statistics was carried out with the DESeq2 software in R environment (13). Negative binomial linear model and Wald test were used to produce *p*-values. Multiple testing adjustment of *p*-values was performed with Benjamini-Hochberg procedure. Basic gene annotation was made with Ensembl Release 99 (14, 15).

### RT-qPCR of Tissue RNA

One and half micrograms (1.5 μg) of total RNA isolated from snap-frozen LV (IHD *n* = 8, Ctrl *n* = 8) were reverse transcribed using SuperScript^TM^ VILO^TM^ cDNA Synthesis Kit (Invitrogen) in a total volume of 20 μl at 42°C for 1 h. The PCR reactions in a total volume of 10 μl were performed using 25 ng of cDNA as a template with LightCycler 480 (Roche) and the QuantiTect SYBR Green PCR Kit (Qiagen). HotStarTaq DNA Polymerase was activated at 95°C for 15 min. Forty cycles were carried out at 94°C for 15 s, 55°C for 30 s, and 72°C for 30 s. Human *NPPA, NPPB, NPPC, MME*, and *ACTB* gene-specific primers were obtained from QuantiTect Primer Assays (Qiagen). Each assay consists of specific forward and reverse primers that are derived from gene sequences contained in the NCBI Reference Sequence database (www.ncbi.nlm.nih.gov/RefSeq). Mann-Whitney U test was used to compare the variables in different groups. *P*-values < 0.05 were considered significant.

### Mass Spectrometry and Proteomics Analysis

Mass spectrometry (MS) analysis was carried out (IHD *n* = 6, Ctrl *n* = 5) at the Proteomics Unit, Viikki, University of Helsinki. About 30–40 mg of LV tissue were homogenized in 8.0 M urea in 50 mM NH_4_HCO_3_ using FastPrep-24 5G bead homogenizer (MP Biomedicals) with 1 mm zirconia beads (BioSpec Products). Total protein concentration was measured with BCA protein assay kit (Pierce, Thermo Scientific) and 50 μg of protein from each sample were taken for the MS analysis. The proteins were reduced with Tris (2-carboxyethyl) phosphine (TCEP; Sigma Aldrich), alkylated with iodoacetamide, trypsin-digested with Sequencing Grade Modified Trypsin (Promega) using 4 μl of trypsin at 37°C for 16 h, and desalted with C18 microspin columns (Nest Group). The desalted samples were recovered in 30 μl of buffer A (0.1% TFA, 1% Acetonitrile in HPLC water) and analyzed on a TripleTOF® 6600 Quadrupole Time-Of-Flight mass spectrometer (Sciex) coupled to an Eksigent nanoLC with a microelectrospray ionization source. YMC-Triart C18 column (12 nm, 3 μm, 150 × 0.3 mm) was used for peptide separation. The MS analysis was performed in the positive-ion mode using information-dependent acquisition (IDA) during a linear 60-min gradient from 5 to 35% buffer B (0.1% formic acid in acetonitrile). Survey scans were acquired in 250 ms and the top 30 ions above the intensity threshold of 150 counts were selected for subsequent MS/MS scans (100–1,500 m/z, 50 ms accumulation time per MS/MS). Raw data were processed with MaxQuant version 1.6.3.4 (16) against the human component of the Uniprot Database (release 01_2020 with 20,303 entries) using the Andromeda search engine (17). Carbamidomethylation of cysteine residues was used as static modification. Aminoterminal acetylation and oxidation of methionine were used as dynamic modification. Trypsin was selected as enzyme, and maximum of two missed cleavages were allowed. Both instrument and label-free quantification parameters were left to default settings. The results were filtered to a maximum false discovery rate (FDR) of 0.05. Differential proteomics was performed using the R packages DEP (differentially enriched or expressed proteins) (18). The data were normalized using variance stabilizing normalization.

### Multi-Omics Integration and Gene Set Analysis

Kinomic, transcriptomic, and proteomic data were integrated by simply concatenating the data together into a single data set. Duplicates between the data sets were removed at random, i.e., Log2FoldChange (LFC) for each gene/protein with duplicates was chosen randomly from the data sets. Hypergeometric testing of integrated data, RNA-seq data, and proteomics data was performed using the clusterProfiler R package (19). Gene set enrichment analysis of RNA-seq and proteomics data was performed using aforementioned packages and any ties in the gene ranks were resolved randomly. Hypergeometric testing was performed using all genes, proteins, and kinases, which had an adjusted *p*-value (BH) < 0.05. Network graphs were produced using igraph (20), and the results were visualized using Cytoscape (21).

## Results

### Patient Characteristics

The clinical characteristics of the IHD patients are summarized in Table 1. A severe reduction of LVEF, ranging from 7 to 32%, was observed in all patients. Seven out of the eight patients had obviously larger LVEDD compared with the recommended size range (male 50.2 ± 4.1 mm; female 45.0 ± 3.6 mm) of normal LV (22). The concentrations of NT-proBNP were markedly increased in the patients despite the fact that they had received conventional medication for heart failure (Table 1), such as β blockers, ACEI, ARB, and diuretics, which are known to decrease plasma levels of NT-proBNP (23, 24).

**Table 1:** Clinical characteristics of patients with IHD.

**Patient ID**	**TX-5**	**TX-14**	**TX-19**	**TX-24**	**TX-25**	**TX-28**	**TX-30**	**TX-34**
Age (years)	58	66	54	52	62	62	53	63
Gender^a^	M	M	M	M	F	M	M	M
BMI (kg/m^2^)	26.1	25.6	29.6	26.5	31.3	26.0	31.0	25.6
Systolic blood pressure^b^ (mmHg)	98	122	115	119	104	110	122	80
Diastolic blood pressure^b^ (mmHg)	61	80	81	83	69	79	77	n/a
LVEDD^c^ (mm)	69	69	71	61	68	65	84	47
LVEF^d^ (%)	23	29	15–20	17	32	27	15	7
NT-proBNP^e^ (pg/ml)	1,562	6,691	6,313	1,278	1,981	1,869	928	2,113
Medications								
β_1_ blocker	+	+					+	
β_1_ and β_2_ blockers					+			
ACEI^f^		+		+		+		
ARB^g^	+							+
ARNI^h^			+		+			+
Diuretics	+	+	+	+	+		+	+
Nitrates	+						+	
*PDE5 blocker*		+		+	+	+	+	+
Pacemaker inhibitor			+					

*a, Gender; M, male; F, female. b, Blood pressure was measured on the day before heart transplantation. c, Left ventricular end-diastolic diameter. d, Left ventricular ejection fraction. e, N-terminal-pro hormone BNP. f, Angiotensin-converting enzyme inhibitor. g, Angiotensin II receptor blocker. h, Angiotensin receptor-neprilysin inhibitor. n/a, not available. +, The corresponding medication was taken by the patient*.

### Phosphorylation of STK-Peptides Was Promoted More Widely and Specifically Than That of PTK-Peptides in IHD Hearts

**Figure 1:**
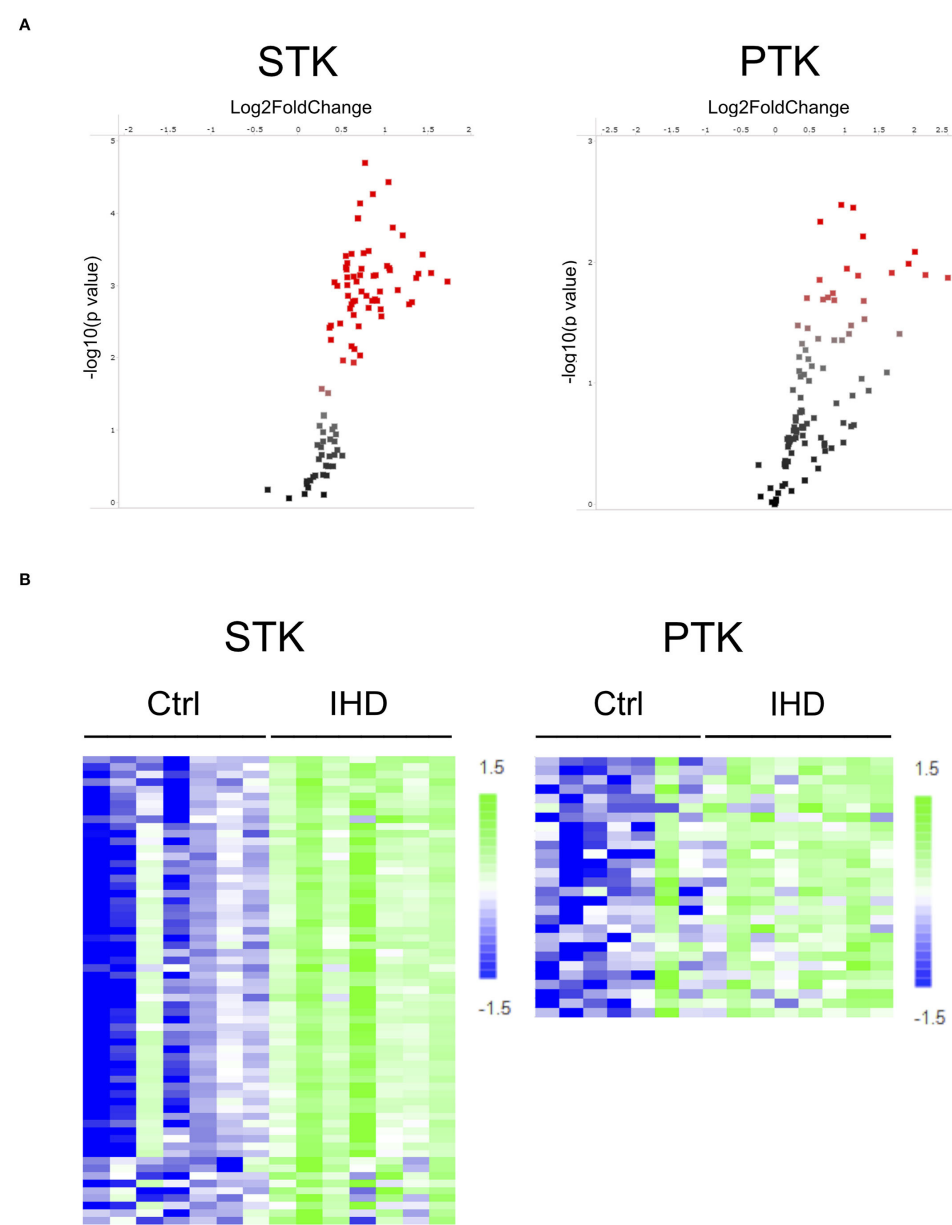
Increased STK- and PTK-peptide phosphorylation in human IHD hearts. **(A)** Volcano plot. Red spots represent peptides having significant difference (*p* < 0.05) between the IHD (*n* = 8) and Ctrl (*n* = 8) groups. **(B)** Scaled heatmap. The increase in phosphorylation of STK- and PTK-peptides was presented by the color change from dark blue in the controls to light green in the IHD patients. The detailed peptide sequences contributing most to the significant changes between the groups are shown in Supplementary Table 2.

To identify difference in protein kinase activity profiles between end-stage IHD LVs and Ctrls, we applied PamChip technology to compare STK and PTK activity on a PamStation12 System. Ninety-six out of 144 STK (serine threonine kinase) peptides showed phosphorylation signals, of which 65.6% (63/96) demonstrated a significant difference between the IHD and Ctrl groups. For PTK (protein tyrosine kinase), 102 out of 199 peptides were phosphorylated, and of those, 27.5% (28/102) showed a significant difference between the groups. The phosphorylation of STK-peptides was enhanced more widely and specifically than that of PTK in the IHD LVs, although a couple of PTKs' activity increased considerably (Figure 1A; Supplementary Table 2). Scaled heatmaps demonstrated the enhanced phosphorylation of the STK- and PTK-peptides in the IHD LVs (Figure 1B). The detailed peptide sequences contributing most to the significant changes between the groups are shown in Supplementary Table 2.

### cAMP/cGMP-Dependent Protein Kinases (PKA and PKG) Ranked on Top Among the Predicted Upstream Kinases

**Figure 2:**
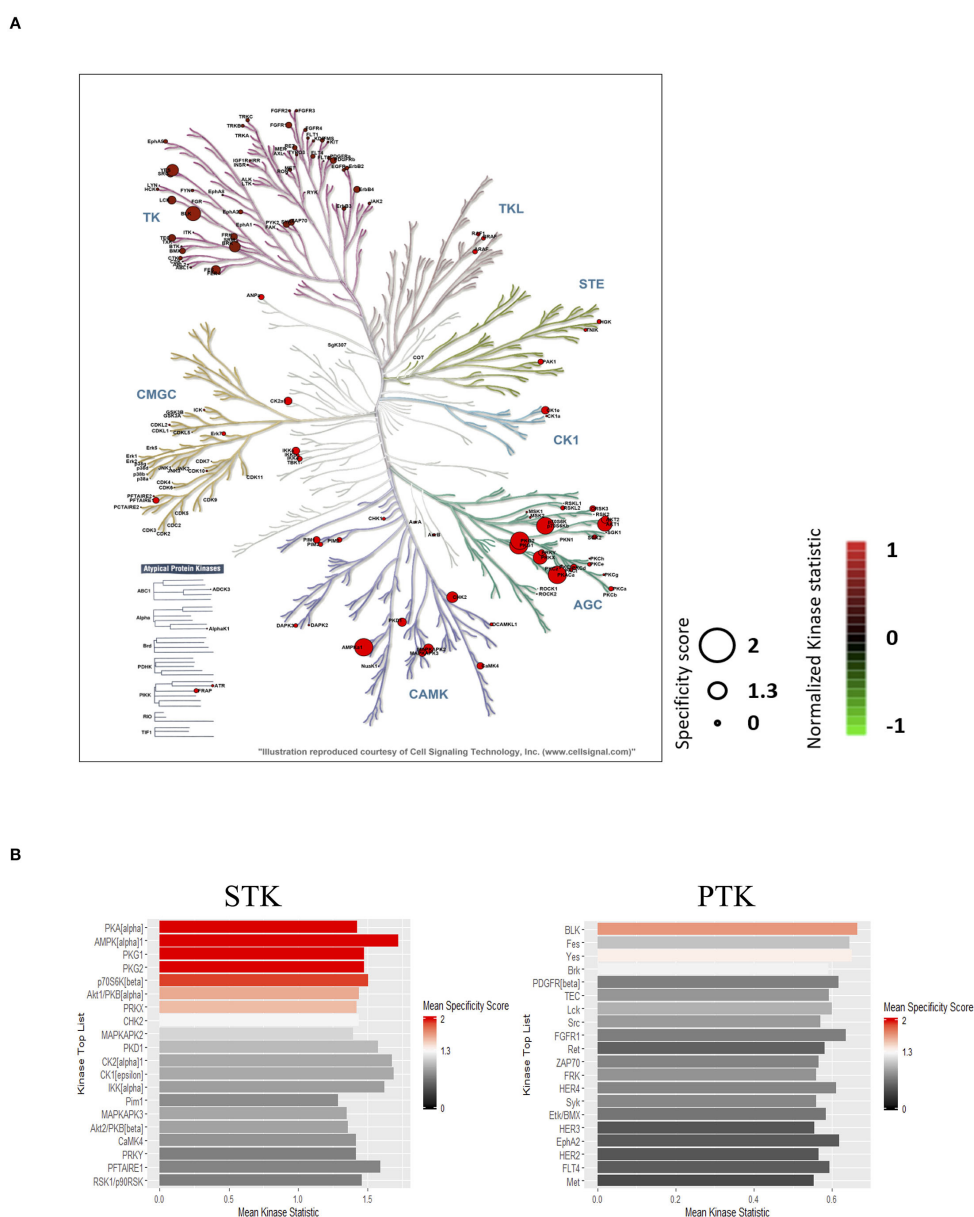
Prediction of the top upstream kinases. **(A)** Combined STK and PTK kinome tree. Top predicted kinases are represented on phylogenetic tree of the human protein kinase family. Dot size indicates specificity score and color denotes kinases statistic (IHD vs. Ctrl). **(B)** Kinase score plot. X-axis (Mean Kinase Statistic) indicates the overall change of the peptide set that represents the kinase. Y-axis (Mean Final Score) ranks kinases based on their significance and specificity in terms of the set of peptides used for the corresponding kinase.

To identify the top kinases responsible for phosphorylating the phosphosites on the PamChip, the Upstream Kinase Analysis tool was applied. Almost all visible normalized kinase statistics showed increased kinase activities in the IHD LVs when compared with controls (Figure 2A). The predicted upstream kinases were scored based on their significance and specificity in terms of the set of peptides used for the corresponding kinase. More specific enhancement was observed in the AGC and CAMK families than those of TK and other families in the human kinome (Figure 2A). The STKs possessed much higher specificity scores than those of PTKs (Figure 2B; Supplementary Table 3). PKA-Cα ranked on top followed by AMPKα1, PKG1, PKG2, p70S6K(β), Akt1/PKB(α), and PRKX. Six out of the top seven STKs belong to the AGC family. Relevant canonical pathway maps were created by Clarivate Analytics® GeneGO Pathway analysis tool. Nociception-nociceptin receptor signaling pathway was identified as the number one involved in the shift of kinase profiling in the IHD LVs. The other top-ranked signaling pathways were mostly related to inflammatory immune responses, which may play essential and deleterious roles in cardiac repair and regeneration (Supplementary Figure 1A). The possible networks were also assembled (Supplementary Figure 1B), displaying complex connections among the various signaling pathways.

### Phosphorylation of PKA-Cα and PKA Activity Decreased Remarkably in IHD Hearts

**Figure 3:**
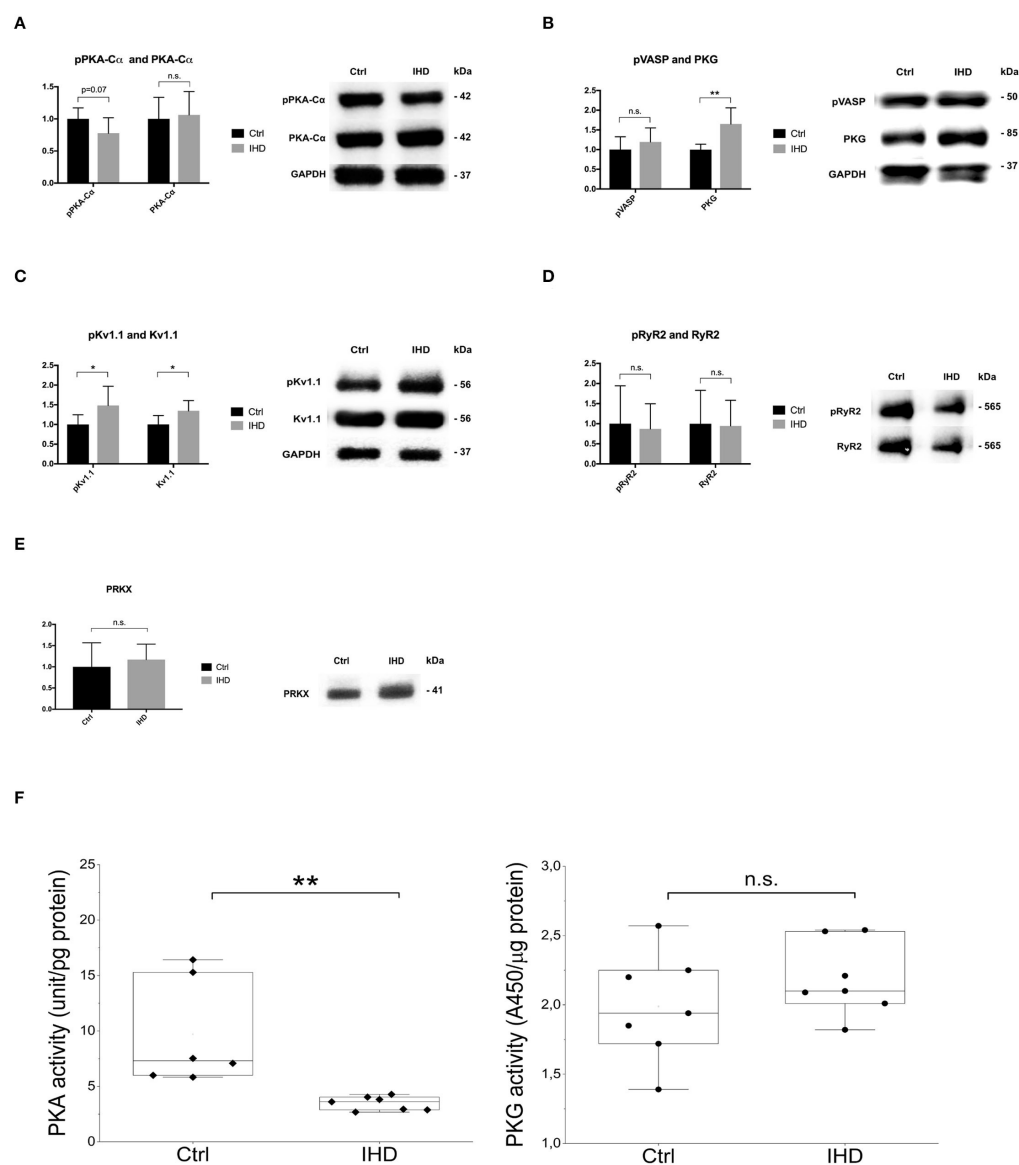
Western blotting and PKA/PKG activity analysis. **(A)** PKA-Cα and phosphorylated PKA-Cα (Thr197). **(B)** PKG1 α and β isoenzymes and phosphorylated downstream target VASP (Ser239). **(C)** Potassium channel Kv1.1 and phosphorylated Kv1.1. **(D)** Ryanodine receptor 2 (RyR2) and phosphorylated RyR2. **(E)** cAMP-dependent protein kinase catalytic subunit PRKX. **(F)** PKA and PKG activity assay. **(A–E)** Total protein loading per lane was used to normalize the sample loading variation (IHD *n* = 7, Ctrl *n* = 7). The error bars represent standard deviation (SD). Only a representative Western blot, from Ctrl and IHD respectively, was presented. **(F)** PKA (IHD *n* = 7, Ctrl *n* = 6) and PKG (IHD *n* = 7, Ctrl *n* = 7) assays were performed in duplicate from 2 different dilution preparations. Statistical analysis was made by *T* tests (and non-parametric tests) in Prism software for Western blotting and Mann-Whitney U test for PKA and PKG activity assay. n.s., not significant, *p < 0.05, **p < 0.01.

To validate the protein kinase profiling results, Western blotting was carried out focusing on PKA-Cα, PKG, Kv1.1, RyR2, and PRKX to determine the protein abundance and phosphorylation levels in the LV tissue. There was an increasing tendency (~5%) in the amount of PKA-Cα in the IHD LVs. However, its phosphorylation reduced substantially (>20%), approaching a statistically significant level when compared with that of controls (*p* = 0.07, Figure 3A). The PKG protein level was remarkably elevated in the IHD group (65%, *p* = 0.003), while the phosphorylation of its downstream target, Ser239 in vasodilator-stimulated phosphoprotein (VASP), was also enhanced (19%), although the increase did not reach statistical significance (*p* = 0.33, Figure 3B). A statistically significant elevation in Kv1.1 (35%, *p* = 0.02) and phosphorylated Kv1.1 (Ser446) (48 %, *p* = 0.037) was observed in the IHD group (Figure 3C). Large variation of RyR2 and phosphorylated RyR2 (S2808) existed in both groups, and no significant difference was found (*p* = 0.89 for RyR2, *p* = 0.77 for pRyR2, Figure 3D). Protein level of PRKX, a PKA-related STK, in the IHD LVs appeared non-significantly increased (14.5%) when compared with the controls (Figure 3E). PKA activity assay from LV lysates showed a highly significant reduction (62%, *p* = 0.0034) in the IHD LVs compared with that of controls, while PKG activity remained stable (Figure 3F).

### RNA-Seq Showed Alterations in Transcription of the Top Predicted Kinases in IHD LVs

To understand the possible changes in the cellular transcriptome and to reveal the presence and quantity in mRNA expression of the top predicted protein kinase genes, RNA-seq analysis was carried out on the IHD LVs (*n* = 8) and compared with the controls (*n* = 7). Significant differential gene expression was found in the regulatory subunits PKA-RIα (*PRKAR1A*: LFC 0.26, padj = 0.045), PKA-RIIα (*PRKAR2A*: LFC−0.32, padj = 0.004), and the catalytic subunit PRKX (*PRKX*: LFC 0.84, padj = 5.19E-06), whereas no significant difference was observed in mRNA levels of the PKA catalytic subunit α, β, and γ (*PRKACA, PRKACB*, and *PRKACG*), and PKG (*PKG1, PKG2)* between the groups (Table 2).

**Table 2:** Differential gene expression of PKA, PKG, and PRKX.

**Gene**	**Description**	**LFC^**a**^**	**padj^**b**^**
PRKACA	Protein kinase cAMP-activated catalytic subunit alpha, PKA C-alpha	−0.03	n.s.^c^
PRKACB	Protein kinase cAMP-activated catalytic subunit beta, PKA C-beta	0.18	n.s.
PRKACG	Protein kinase cAMP-activated catalytic subunit gamma, PKA C-gamma	−0.45	n/a^d^
PRKAR1A	Protein kinase cAMP-dependent type I regulatory subunit alpha	0.26	0.045*
PRKAR1B	Protein kinase cAMP-dependent type I regulatory subunit beta	−0.14	n.s.
PRKAR2A	Protein kinase cAMP-dependent type II regulatory subunit alpha	−0.32	0.004**
PRKAR2B	Protein kinase cAMP-dependent type II regulatory subunit beta	0.33	n.s.
PRKG1	Protein kinase cGMP-dependent 1, PKG1	−0.04	n.s.
PRKG2	Protein kinase cGMP-dependent 2, PKG2	−0.33	n.s.
PRKX	Protein kinase cAMP-activated catalytic subunit PRKX	0.84	5.2E-06*****

a *Log2FoldChange*.

b Adjusted p values < 0.05 are regarded as statistically significant. *padj < 0.05, **padj < 0.01, *****padj < 0.00001.

c *n.s., not significant*.

d *n/a, not available*.

mRNA levels of MAPK activated protein kinase 2 (*MAPKAPK2*) and 3 (*MAPKAPK3*), protein kinase B β (*AKT2*), and platelet-derived growth factor receptor β (*PDGFRB*) decreased significantly, while that of BMX (*BMX*) increased significantly in the IHD group (Supplementary Table 4). These genes have a wide spectrum of functions, including regulation of cell proliferation and differentiation, promotion of cell survival, and modulation of cellular metabolism.

### Differential Gene Expression in cAMP/PKA and cGMP/PKG Signaling Pathways

#### mRNA Levels of the Enzymes Responsible for Synthesis and Hydrolysis of cAMP Altered Significantly

The cAMP and cGMP maintain physiological cardiac contractility and integrity. To gain better knowledge on the differential gene expression of the enzymes responsible for cAMP/cGMP synthesis and hydrolysis, the mRNA levels of those proteins were investigated. Four adenylate cyclase genes (*ADCY1, 3, 5, 9*) were significantly downregulated under IHD condition, while no significant difference was detected between the groups among mRNAs encoding for guanylate cyclases (*GUCYs*) and the natriuretic peptide receptors (*GUCY2A, GUCY2B*, and *NPR-3*) (Table 3). The data suggest that the synthesis of cAMP could be strongly affected by reduced adenylate cyclase gene expression, whereas cGMP synthesis might not be affected significantly. Differential gene expression in the cyclic nucleotide-hydrolyzing phosphodiesterases (PDEs) was found to be bidirectional (Table 3). The mRNA levels of *PDE4A* (cAMP-specific, LFC −1.18, padj = 3.011E-12), *PDE4D* (cAMP-specific, LFC −0.68, padj = 0.02), and *PDE1C* (cAMP/cGMP dual specificity, LFC−0.55, padj = 0.006) decreased significantly, whereas those of *PDE3B* (cAMP/cGMP dual specificity, LFC 0.78, padj = 0.003) and *PDE7A* (cAMP-specific, LFC 0.72, padj = 8.6E-04) increased significantly in the IHD group. The most significant decrease was observed in *PDE4A* mRNA, which hydrolyzes cAMP specifically. No difference was found in the gene expression of *PDE5A* (Table 1).

**Table 3:** Differential gene expression in cAMP/cGMP synthesis and hydrolysis.

**Gene**	**Description**	**LFC^**a**^**	**padj^**b**^**
ADCY1	Adenylate cyclase 1	−0.83	1.4E-04***
ADCY2	Adenylate cyclase 2	0.83	n.s.^c^
ADCY3	Adenylate cyclase 3	−0.82	0.003**
ADCY4	Adenylate cyclase 4	0.27	n.s.
ADCY5	Adenylate cyclase 5	−0.59	0.01*
ADCY6	Adenylate cyclase 6	−0.07	n.s.
ADCY7	Adenylate cyclase 7	0.49	n.s.
ADCY8	Adenylate cyclase 8	−0.52	n/a^d^
ADCY9	Adenylate cyclase 9	−0.49	2.7E-06*****
ADCY10	Adenylate cyclase 10	0.37	n.s.
GUCY1A1	Guanylate cyclase 1 soluble subunit alpha 1	−0.09	n.s.
GUCY1A2	Guanylate cyclase 1 soluble subunit alpha 2	−0.20	n.s.
GUCY1B1	Guanylate cyclase 1 soluble subunit beta 1	−0.20	n.s.
GUCY1B2	Guanylate cyclase 1 soluble subunit beta 2	1.47	n.s.
GUCY2A	Guanylate cyclase A, Natriuretic peptide receptor 1, NPR-A	0.18	n.s.
GUCY2B	Guanylate cyclase B, Natriuretic peptide receptor 2, NPR-B	0.08	n.s.
NPR3	Natriuretic peptide receptor 3, NPR-C	0.51	n.s.
GUCY2C	Guanylate cyclase 2C, Guanylate cyclase C	−0.40	n.s.
GUCY2D	Guanylate cyclase 2D, Retinal	0.52	n/a
GUCY2EP	Guanylate cyclase 2E, Pseudogene	1.00	n/a
GUCY2F	Guanylate cyclase 2F, Retinal	−1.26	n/a
GUCY2GP	Guanylate cyclase 2G, Pseudogene	−0.09	n/a
PDE1A	Phosphodiesterase 1A	−0.87	n.s.
PDE1B	Phosphodiesterase 1B	−0.13	n.s.
PDE1C	Phosphodiesterase 1C	−0.55	0.006**
PDE2A	Phosphodiesterase 2A	0.34	n.s.
PDE3A	Phosphodiesterase 3A	−0.29	n.s.
PDE3B	Phosphodiesterase 3B	0.78	0.003**
PDE4A	Phosphodiesterase 4A	−1.18	3.0E-12*****
PDE4B	Phosphodiesterase 4B	−0.37	n.s.
PDE4C	Phosphodiesterase 4C	0.31	n.s.
PDE4D	Phosphodiesterase 4D	−0.68	0.02*
PDE5A	Phosphodiesterase 5A	0.07	n.s.
PDE6A	Phosphodiesterase 6A	1.01	0.05
PDE6B	Phosphodiesterase 6B	−0.02	n.s.
PDE6C	Phosphodiesterase 6C	0.32	n/a
PDE6D	Phosphodiesterase 6D	0.02	n.s.
PDE7A	Phosphodiesterase 7A	0.72	8.6E-04***
PDE7B	Phosphodiesterase 7B	−0.29	n.s.
PDE8A	Phosphodiesterase 8A	0.12	n.s.
PDE8B	Phosphodiesterase 8B	0.66	n.s.
PDE9A	Phosphodiesterase 9A	0.27	n.s.
PDE10A	Phosphodiesterase 10A	−0.36	n.s.
PDE11A	Phosphodiesterase 11A	−0.81	n.s.
PDE12	Phosphodiesterase 12	−0.01	n.s.

a *Log2FoldChange*.

b Adjusted p values < 0.05 are regarded as statistically significant. *padj < 0.05, **padj < 0.01, ***padj < 0.0001, *****padj < 0.00001.

c *n.s., not significant*.

d *n/a, not available*.

#### The Most Altered Gene Expression Was Observed in Natriuretic Peptide Genes

**Figure 4:**
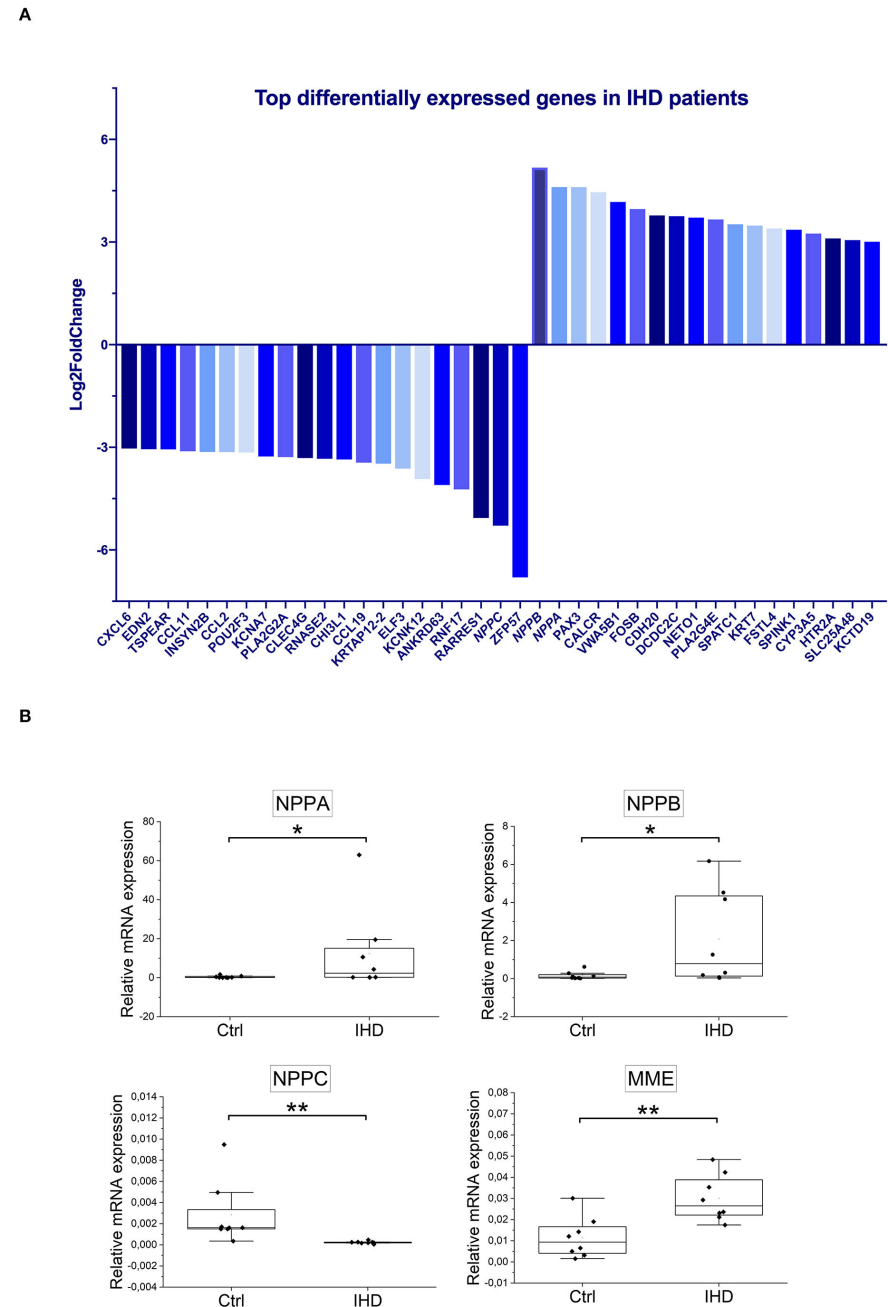
Top differentially expressed genes in IHD patients. **(A)** The significantly altered genes were ranked based on Log2FoldChange (IHD *n* = 8, Ctrl *n* = 7). Only the ones with values ≥±3 were displayed. **(B)** Tissue RT-qPCR validation (IHD *n* = 8, Ctrl *n* = 8) for *NPPA, NPPB, NPPC*, and *MME* genes. *ACTB* (β-actin) was taken as an internal control. Statistical analysis was made by Mann-Whitney U Test. *P* < 0.05 was considered as statistically significant. n.s. not significant, *p < 0.05, **p < 0.01.

In RNA-seq analysis, 932 protein coding mRNAs were significantly upregulated (Supplementary Table 5) and 1,485 were downregulated (Supplementary Table 6) in the IHD LVs when compared with controls. The most enhanced mRNA expression under IHD condition was seen in natriuretic peptide B (*NPPB*) and A (*NPPA*) genes with LFC values of 5.2 (padj = 4.3E-06) and 4.6 (padj = 0.0019), respectively. The mRNA level of natriuretic peptide C gene (*NPPC*) decreased sharply with LFC value at −5.3 (padj = 2.9E-11) (Figure 4A). Tissue RT-qPCR was performed to validate the transcript alterations. Neprilysin gene (*MME*), responsible for natriuretic peptide degradation, was also tested due to its significant elevation in the patients (Supplementary Table 5, LFC 1.25, padj = 0.0096). The results were consistent with the RNA-seq data showing that mRNA levels of *NPPA* (padj = 0.036), *NPPB* (padj = 0.046), and *MME* (padj = 0.006) increased significantly under IHD condition, whereas that of *NPPC* (padj = 0.001) decreased remarkably when compared with controls (Figure 4B).

#### Extracellular Matrix Remodeling and G Protein-Coupled Receptor Signaling Were Highlighted in Gene Ontology Enrichment Analysis

**Figure 5:**
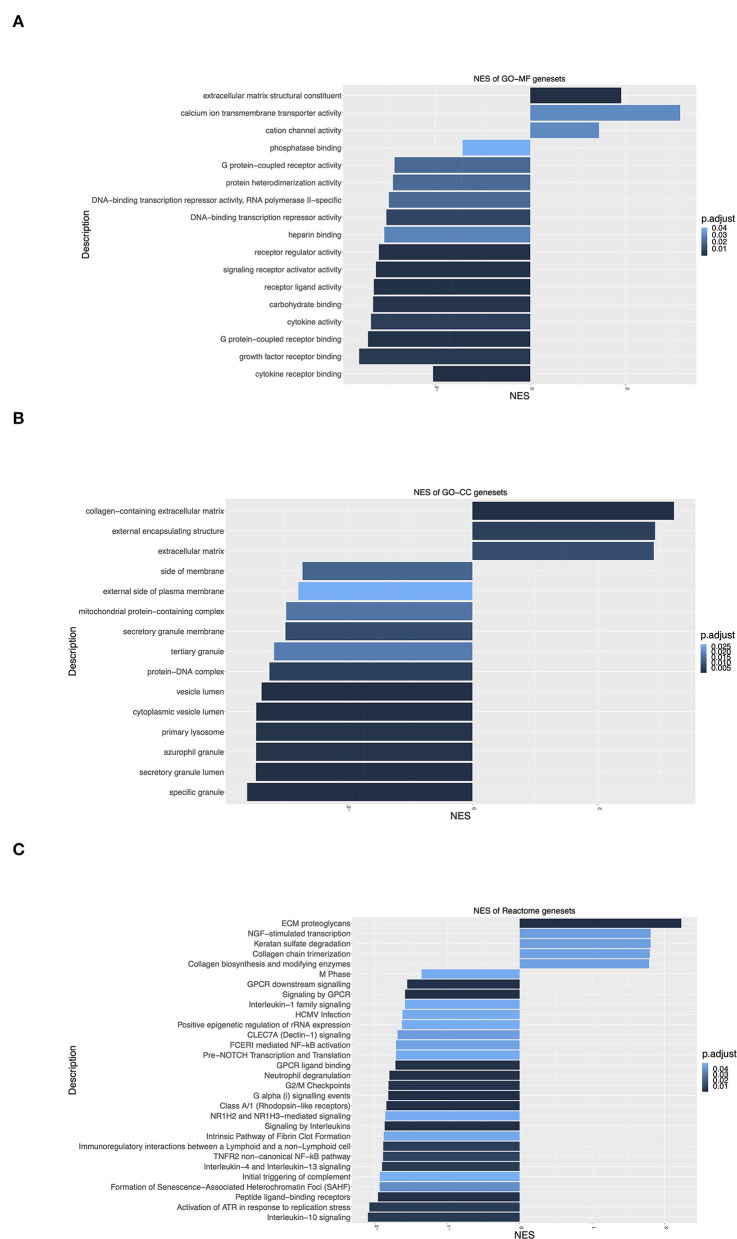
Gene Ontology (GO) enrichment analysis of differentially expressed genes. **(A)** Molecular Function (GO-MF) enrichment. **(B)** Cellular Component (GO-CC) enrichment. **(C)** Reactome signaling pathway enrichment. Y-axis label represents different GO terms, and X-axis label represents normalized enrichment scores (NES) (IHD *n* = 8, Ctrl *n* = 7). Positive/negative enrichment scores indicate a shift of genes toward upregulation/downregulation of the ranked list. Adjusted *p*-value (BH) or p.adjust < 0.05 means significant change.

To identify groups of genes that share common biological function or regulation, and show statistically significant, concordant differences between IHD and Ctrl, Gene Ontology (GO) enrichment analyses were performed. The hallmark change with positive normalized enrichment score (NES) was seen in the clusters of genes related to “Extracellular matrix structural constituent” (Figure 5A), “Collagen-containing extracellular matrix,” “External encapsulating structure,” “Extracellular matrix” (Figure 5B), and “ECM proteoglycans” (Figure 5C). The data clearly reflected the enhanced cardiac remodeling in the patients with IHD. Signaling by anti-inflammatory interleukins IL-10, IL-4, and IL-13 changed significantly with a negative NES in the patients (Figure 5C). These interleukins are well-known to suppress immune responses and augment tissue remodeling during infection and tissue repair. Significant negative NES values were also observed in the genes related to Gα(i) signaling events, GPCR ligand binding, signaling by GPCR, and GPCR downstream signaling (Figure 5C), implying that GPCR signaling pathways played a key role in the kinase activity profile shift in the IHD hearts.

### Proteomics Analysis Revealed Significantly Reduced Protein Levels in Energy Metabolism and Muscle Contraction in IHD Hearts

**Figure 6:**
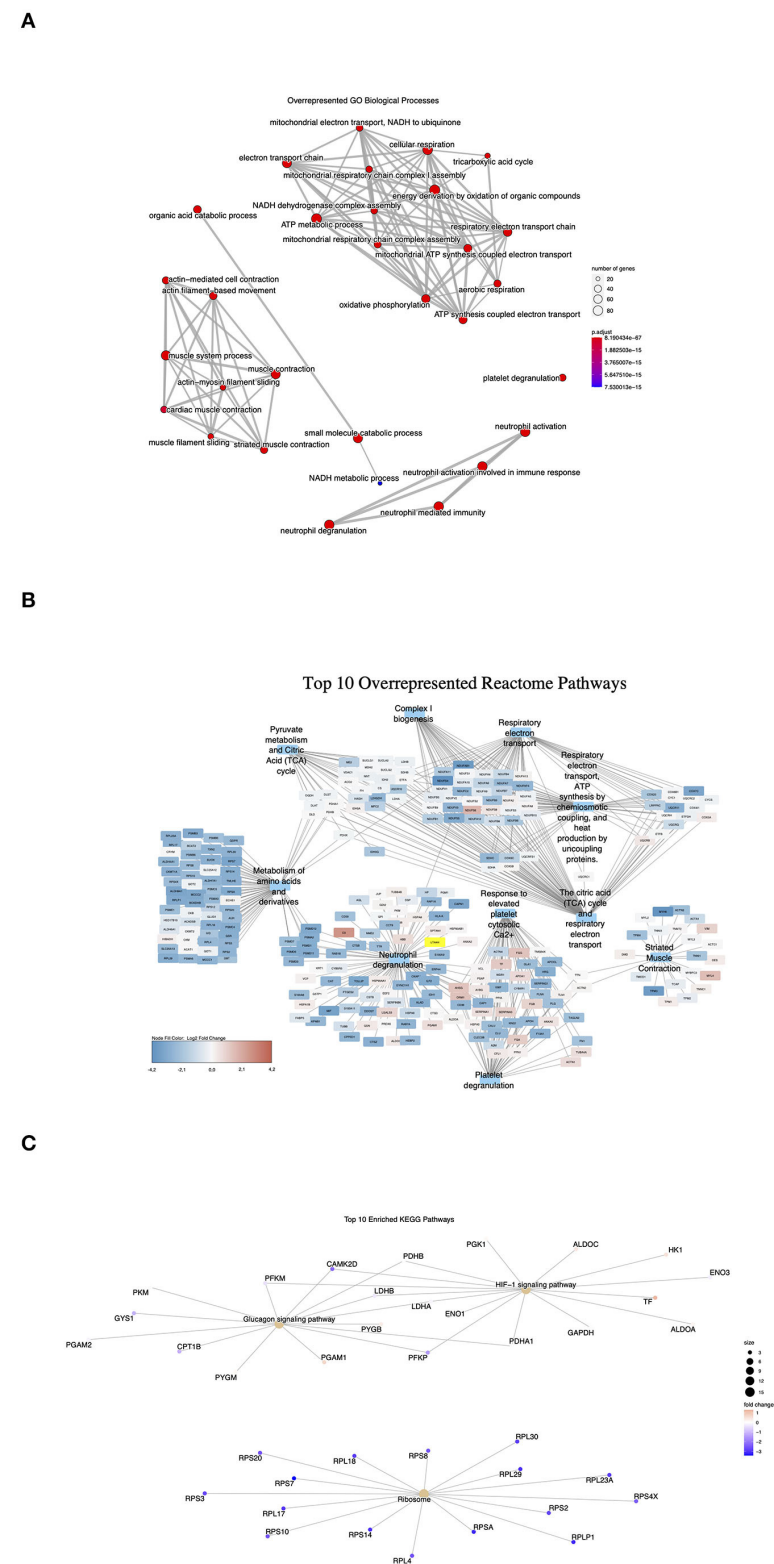
Overrepresented signaling pathways in proteomics analysis. Dot size represents the number of protein encoding genes enriched in each pathway (IHD *n* = 6, Ctrl *n* = 5). **(A)** GO Biological processes. Adjusted *p*-value (p.adjust): red < purple < blue. **(B)** Top 10 Reactome pathways. **(C)** Enriched KEGG pathways. Red color represents upregulated protein levels. Purple and blue colors represent downregulated protein levels.

To investigate the effects of differential gene expression at the protein level, mass-spectrometry (MS)-based proteomics analysis was carried out on the LV tissue lysates (IHD *n* = 6, Ctrl *n* = 5). A total of 829 differentially expressed proteins were identified between the IHD and Ctrl LVs. Ninety-four proteins reached a statistically significant level (padj < 0.05), being all reduced in the IHD hearts compared with controls (Supplementary Table 7). Two major protein groups were identified in hypergeometric testing (Figure 6A). The proteins were associated with cardiac energy metabolism and muscle contraction, which covered not only respiratory electron transport, energy metabolism and citric acid cycle, metabolism of amino acids and derivatives, but also striated muscle contraction, neutrophile, and platelet degranulation (Figure 6B). Overall, the differentially expressed proteins are closely related to cardiac energy metabolism, muscle contractility, inflammatory responses, and thrombin activation. Hypoxia-inducible factor 1 signaling pathway, ribosome biogenesis, and glucagon signaling pathway were the top three highlighted under IHD condition in KEGG gene set enrichment analysis (Figure 6C). The data revealed the body's adaptation response to hypoxic conditions through transcriptional activation of many genes including ones involved in glucose metabolism.

### Multi-Omics Integration Analysis Highlighted Intracellular Signaling by Second Messengers as the Top Reactome-Enriched Pathway

To further understand the overall profile shift of kinases in IHD LVs, we integrated kinomic, transcriptomic, and proteomic datasets to uncover the common functional context of the multi-omics features. These functional contexts refer to different signaling pathways or biological processes in which features from multi-omics were enriched. A total of 66 Reactome-enriched pathways were significantly affected (Supplementary Table 8). “Intracellular signaling by second messengers” was the most significantly altered pathway followed by “Signaling by interleukins” (Table 4). “cGMP-PKG signaling pathway” and “cAMP signaling pathway” were ranked within the top 20 among the total 105 significantly altered KEGG-enriched signaling pathways (Table 4; Supplementary Table 9). MAPK family signaling cascades and PI3K-Akt signaling pathway were also highlighted in both Reactome and KEGG enrichment analysis (Table 4). “Cellular response to peptide,” “Response to peptide hormone,” “Response to oxygen levels,” “Regulation of actin filament-based process,” and “Response to decreased oxygen levels” were highlighted among the total 407 significantly changed biological processes in GO Biological Process enrichment analysis (Supplementary Table 10).

**Table 4:** Top 10 signaling pathways and biological processes in integration analysis.

**Ranking**	**Pathway**	***p*-value**	**padj**
**Reactome (/66)**			
1	Intracellular signaling by second messengers	1.81E-08	2.40E-05
2	Signaling by Interleukins	4.38E-08	2.40E-05
3	FLT3 Signaling	5.35E-08	2.40E-05
4	MAPK family signaling cascades	1.92E-07	6.46E-05
5	MAPK1/MAPK3 signaling	2.59E-07	6.46E-05
6	RAF/MAP kinase cascade	2.88E-07	6.46E-05
7	Diseases of signal transduction by growth factor receptors and second messengers	1.47E-06	2.82E-04
8	Interleukin-37 signaling	1.71E-06	2.88E-04
9	PI3K/AKT signaling in cancer	2.22E-06	3.32E-04
10	Interleukin-1 family signaling	3.69E-06	4.97E-04
**KEGG (/105)**			
1	PI3K-Akt signaling pathway	6.54E-13	2.05E-10
2	Insulin signaling pathway	2.83E-12	4.44E-10
3	MAPK signaling pathway	6.20E-12	6.47E-10
4	Ras signaling pathway	8.53E-09	6.68E-07
5	EGFR tyrosine kinase inhibitor resistance	3.82E-08	2.39E-06
6	Focal adhesion	1.05E-07	5.47E-06
7	Rap1 signaling pathway	1.45E-07	6.50E-06
8	Prostate cancer	2.17E-07	8.49E-06
9	cGMP-PKG signaling pathway	2.77E-07	9.62E-06
10	Neurotrophin signaling pathway	5.00E-07	1.57E-05
**GO-BP (/407)**			
1	Ras protein signal transduction	1.72E-13	1.03E-09
2	Cellular response to peptide	1.28E-11	3.85E-08
3	Regulation of small GTPase mediated signal transduction	2.73E-11	5.46E-08
4	Response to peptide hormone	1.24E-10	1.86E-07
5	Epithelial tube morphogenesis	6.05E-10	7.26E-07
6	Rho protein signal transduction	8.05E-10	8.04E-07
7	Response to oxygen levels	1.04E-09	8.92E-07
8	Regulation of actin filament-based process	1.20E-09	9.02E-07
9	Urogenital system development	1.39E-09	9.25E-07
10	Response to decreased oxygen levels	2.03E-09	1.22E-06

## Discussion

To the best of our knowledge, this is the first study to profile the cardiac kinase activity in human end-stage IHD and to investigate the shift of the related signaling pathways by multi-omics approach. PKA and PKG ranked on top among the altered kinases. PKA activity in the IHD LVs reduced remarkably, though with significant upregulation of PRKX and downregulation of PKA-RIIα genes. mRNA levels of adenylate cyclase (*ADCY 1, 3, 5, 9*) and cAMP-hydrolyzing phosphodiesterase (*PDE4A, PDE4D*) decreased significantly. No statistically significant alterations were observed from either the PKG activity or the mRNA levels of PKG and guanylate cyclase. However, mRNA expression of CNP gene decreased remarkably, whereas those of BNP, ANP, and neprilysin genes increased significantly. The findings suggest that the deficiency in cAMP/PKA signaling pathway could contribute considerably to the pathogenesis of IHF. The significant decrease in *ADCY* gene expression may cause insufficient cAMP synthesis, and a major PKA inactivation, which leads to a dominant reduction in cardiac energy metabolism and muscle contractility under the basis that the hearts have suffered from long-term inflammation and cardiac remodeling. The natriuretic peptide CNP, in addition to ANP, BNP, and neprilysin, could also be a potential target when attempting to modulate the cGMP/PKG signaling pathway as part of a pharmacotherapeutic regimen in the treatment of IHF.

The cyclic nucleotide second messengers, mainly cAMP and cGMP, and their downstream STKs play a multiple role in vessel physiology and homeostasis, and in the pathogenesis of cardiovascular disorders. As emerging and key controllers in cardiovascular disease pathology, the cyclic nucleotide driven AGC family of STKs, especially PKA and PKG, draw particular attention. PKA is kept inactive when forming a complex of two catalytic and two regulatory subunits. Ligands such as adrenaline bind and activate GPCRs, leading to the Gα to associate with adenylate cyclase and produce cAMP that binds to the PKA-RI or PKA-RII. The binding induces release of the active PKA-C and allows them to phosphorylate substrates (25). Significant downregulation in 4 out of 10 adenylate cyclases explains, at least partially, the striking reduction of PKA activity in the IHD LVs. It can be speculated that cAMP synthesis decreased, leading to less binding to PKA-R, less activation of PKA-C, and consequently deficiency in PKA signaling. Surprisingly, no significant change in the mRNA levels of PKA-Cα/β/γ and the tendentious increase in PKA-Cα protein suggest that the low PKA activity is not due to reduced PKA-C production. Furthermore, no significant increase was seen in mRNA levels of PKA inhibitors, e.g., PKI β and γ, in cAMP-independent pathway, and even a significant decrease in mRNA level of PKI α was found (data not shown), indicating that the inactivation of PKA did not result from the presence of robust protein kinase inhibitors either. Significant upregulation of PKA-RIα gene expression implies that a slow disassociation of PKA-C from the holoenzyme may exist in the IHD LVs, contributing additionally to the inactivation of PKA. Phosphorylation of PKA-C (Thr-197) is essential for its maturation and optimal biological function (26). The decreased phosphorylation in PKA-C (Thr-197) could also play a role in PKA inactivation. It remains elusive whether there is an *in vivo* regulatory mechanism involving phosphorylation and dephosphorylation of Thr-197 in humans. In the current study, low PKA activity could not stimulate upstream adenylate cyclase activation and cAMP production, indicating that the feedback loop of cAMP/PKA signaling might be disrupted. It could be partially due to the β blocker medication in the patients. The mechanism underlying the action of β blockers in IHF is poorly understood. The negative inotropic effects of β blockers can be problematic. The same holds true for ACEI and diuretic therapy as well. Therefore, they are limited by initiating with small doses, uptitrating gradually, and monitoring heart rate, blood pressure, and clinical status after each dose increase (27). Our data have provided evidence to support the emerging ideas to selectively targeting cAMP modulators or effectors as innovative curatives for cardiac diseases including IHD (28). PRKX is related but distinct from the PKA-C (25) and activated at lower cAMP concentration than the PKA holoenzyme (29). PKA-RIα preferentially binds to PRKX *in vivo* and PKA-RIIα is a potent inhibitor of PKA-C but not of PRKX (29). The mechanisms underlying the activation of PRKX are still largely unknown (25, 30). Although elevations in the mRNA (LFC 0.84, padj = 5,192E-06) and protein (14.5%) levels were found, no direct compensation of PKA activity by PRKX was observed in the IHD LVs. Nevertheless, PRKX can modulate the cAMP-mediated signal transduction by binding to the RIα and by phosphorylating the RIIα in the absence of cAMP (29). It is very likely that the increased PRKX may compete for PKA-C in binding to RIα or may enhance the phosphorylation of RIIα and greatly reduce the reassociation with the released PKA-C, thereby prolonging its activation. It should be noted that the compartment-specific loss of PKA and the local PKA activity compensation cannot be ruled out, as subcellular localization of PKA is essential for proper signaling.

Protein kinase G exists in two isoforms that are regulated by cGMP. Natriuretic peptides (NPs) and nitric oxide activate, respectively, particulate (pGCs) and soluble (sGCs) guanylate cyclases, thereby modulating the cellular cGMP levels and the downstream PKG activity. The strong cardiac upregulation of PKG may play important roles in the attenuation of pathological cardiac hypertrophy and remodeling (31). Phosphorylation and dephosphorylation of its downstream target VASP are important functional regulatory mechanisms that affect the actin cytoskeletal dynamics and further muscle contraction. Understanding these patterns allows for defining the pathological mechanisms responsible for disturbances in actin organization and disease development. Natriuretic peptides ANP and BNP are considered as the main endocrine guardians of cardiac function and blood pressure, while CNP has been less focused. cGMP production by CNP/pGC stimulation has been shown to be greater and remain elevated longer than that due to nitric oxide/sGC stimulation in human endothelial cells from umbilical vein (HUVEC) (32). Accumulating evidence has revealed diverse endogenous roles of CNP, e.g., in control of vascular tone and integrity, angiogenesis, anti-fibrosis, and cardioprotection (33, 34). The remarkable reduction in CNP gene expression in the IHD LVs may explain partially the enhanced cardiac remodeling. All NPs are cleared by NPR-C and neprilysin (35). The significant upregulation of neprilysin gene in cardiac LV in the patients could be partially responsible for the striking cardiac mRNA upregulation of BNP and ANP, as the enhanced degradation could be a positive feedback signal for enhanced NP production. Furthermore, the remarkable upregulation of BNP and ANP could also promote the activation of NPR-A receptor, and thus more cGMP production. This may explain the significantly elevated PKG protein level observed in the IHD LVs, though the increase of PKG activity was only approximately 10%. There have been contradictory data about the CNP gene expression in end-stage failing hearts (36, 37). Dysregulation of the NP system exists in human cardiovascular diseases. It is well known that at symptomatic HFrEF, not only pro-BNP but most importantly, pro-ANP cleavage are impaired, and release of physiologically active ANP/BNP is compromised, despite profound increases in cardiac expression levels of pro-ANP/pro-BNP and plasma levels of immunoreactive ANPs/BNPs forms (38–41).

The PDEs are the key regulators of the cAMP/cGMP signaling pathways in the heart and essential for mediating crosstalk between them (42). The majority of cardiac cyclic nucleotide hydrolysis is mediated by PDE1 in humans, but its role in cardiac physiology is largely unknown. In this study, mRNA levels of all *PDE1* subtypes in the IHD group decreased with *PDE1C* reaching statistical significance. *PDE1C* deletion has an anti-fibrotic effect and PDE1 inhibition improves cardiac function in failing mouse hearts (43). Pharmacological and genetic ablation of *PDE1* has strengthened protective cAMP signaling in larger mammals, enhancing inotropy, vasodilation, and limiting apoptosis (44, 45). mRNA levels of *PDE4A* and *PDE4D* decreased significantly in the patients, suggesting that the specific degradation of cAMP by PDE4 was slowing down to maintain cAMP/PKA signaling. PDE3 hydrolyzes cAMP and cGMP. When it binds to cGMP, cAMP metabolism becomes less active. Thereby, cGMP serves as a positive regulator of cAMP signaling through PDE3, placing it as a critical regulator of cAMP and cGMP crosstalk (42). PDE3 has also been distinctively controlled by different NPs. CNP/cGMP improves the positive inotropic and lusitropic responses to β-AR stimulation in isolated rat cardiomyocytes *via* PDE3 antagonism, whereas BNP/cGMP does not (46). Inhibition of PDE3 activity by cGMP may be more important at low cAMP concentration. This agrees with the findings that cAMP/PKA signaling was overall weakened in the IHD patients. It is very likely that the effects of cAMP induced by the upregulation of *PDE3B* would actually depend on the subcellular cGMP concentration generated by different NPs.

The cAMP/PKA and cGMP/PKG signaling pathways are in a complex network with a precise spatial coordination. It is of high importance to understand how they are interlinked, and how the interactions between NPs and cAMP-/cGMP-mediated signaling pathways and their crosstalk may affect the physiological/pathophysiological processes. cGMP-mediated regulation of cAMP signaling has functional implications because it influences protein phosphorylation downstream of PKA and cardiomyocyte contractility. The modulation of cAMP levels by cGMP may offer a new possibility to selectively regulate local cAMP signals to ameliorate the efficacy of the treatment of ischemic cardiovascular disorders, particularly the IHF.

### Strengths and Limitations

Cardiac kinases play a critical role in cardiac adaptation to stress, and in the progression of IHD. Little systematic investigations of cardiac kinome exist (3, 4). The previous studies mainly focused on kinase expression profiling by microarrays and the kinase proteome by mass spectrometry without global cardiac transcriptome and proteome analysis. To the best of our knowledge, this is the first study to profile the cardiac kinase activity and to carry out global profiling of cardiac transcriptome and proteome, as well as to study the alterations of related signaling pathways by an integrated approach. Furthermore, human LVs were used in our study. Most of the cardiovascular omics studies have been carried out using *in vivo* mouse models, *in vitro* cell cultures, or plasma samples from cardiovascular disease patients (7). Discoveries from human studies have better translational potential than animal models, though both provide important insight into the disease. The use of cardiomyocytes in studies also has limitations due to the complex nature of IHD caused by multiple cell types. Fibroblasts and endothelial cells comprise a small proportion of the cardiac tissue; however, they may determine much of the cardiac mRNA content, while cardiomyocytes might contribute the most to the cardiac protein pool (7). Moreover, RNA-Seq was employed to conduct transcriptional profiling in our study, offering higher specificity and sensitivity to detect a significantly larger number of differentially expressed genes and affected pathways compared with traditional microarray platforms (3, 4, 47). This study has several limitations. Most importantly, only a small number of patients were studied due to limited sample availability. The relatively small sample size makes it difficult to assess potential important population differences between IHD and Ctrl. Moreover, variation exists in plasma NT-proBNP levels and potentially important medications between the patients, which may have an impact on the data accuracy. The generalizability of our findings to the IHD patient population requires further study. Validation of the results should be conducted in a higher number of patients in the future. In addition, other variables such as the comorbidity, multiethnicity, gender, and age should also be taken into consideration. Lastly, samples isolated from specialized nanodomains might be better than the ones from whole tissue lysates, because individual PKA target proteins may be selectively activated *via* highly localized cAMP levels and protected from those in the bulk cytosol.

In summary, ischemic heart failure is a complex disease composed of multiple pathophysiological processes. Although a borderline between the cause and the consequence in this study cannot be drawn clearly, PKA and PKG were highlighted as major findings in the cardiac kinase activity profiling in the patients with IHD. Integrated approach of the multi-omics data revealed that the deficiency in intracellular signaling by second messenger, i.e., cAMP/PKA signaling pathway, may contribute considerably to the pathogenesis of IHD. In addition to ANP, BNP, and neprilysin, CNP could play a role in modulation of cGMP/PKG signaling pathways *via* CNP/pGC/cGMP route, thus representing a potential therapeutic target for the treatment of IHD. However, further investigation is required to address this issue.

## Data Availability Statement

The RNA-seq dataset has been deposited in Gene Expression Omnibus (GEO, https://www.ncbi.nlm.nih.gov/geo/ and is available under GSE203160).

## Ethics Statement

The investigation conformed to the Declaration of Helsinki. The Ethics Committee of Helsinki and Uusimaa Hospital District approved the study. The National Authority for Medicolegal Affairs approved the usage of tissues from organ donors. The patients/participants provided their written informed consent to participate in this study.

## Author Contributions

CW, IT, and PL: conceptualization. CW, JTa, HS, KI, RK, JTo, MM, JS, and PL: methodology. CW, JTa, HS, KI, RK, JTo, MM, PK, VO, JS, ML, IT, and PL: investigation and review and editing. CW, JTa, KI, RK, IT, and PL: original draft preparation. CW and PL: supervision. PL and IT: project administration and funding. All authors contributed to the article and approved the submitted version.

## Funding

This study was supported by grants from the Finnish Cultural Foundation (PL), the Finnish Foundation for Cardiovascular Research (IT and PL), Aarne Koskelo Foundation (IT and PL), the Finnish Foundation for Laboratory Medicine (PL), Finska Läkaresällskapet (IT), the Liv och Hälsa Foundation (IT and PL), the Finnish Society of Clinical Chemistry (PL), Finnish state funding for university-level research (TYH2019320, IT; TYH2021114, PL), and the Finnish Medical Foundation (MM). Wihuri Research Institute is maintained by the Jenny and Antti Wihuri Foundation (PK). The funders had no role in study design, data collection and analysis, decision to publish, or preparation of the manuscript.

## Conflict of Interest

The authors declare that the research was conducted in the absence of any commercial or financial relationships that could be construed as a potential conflict of interest.

## Publisher's Note

All claims expressed in this article are solely those of the authors and do not necessarily represent those of their affiliated organizations, or those of the publisher, the editors and the reviewers. Any product that may be evaluated in this article, or claim that may be made by its manufacturer, is not guaranteed or endorsed by the publisher.

## Abbreviations

IHD: ischemic heart disease
LV: left ventricular/ventricle
STK: serine threonine kinase
PTK: protein tyrosine kinase
cAMP: cyclic adenosine 3' 5'-monophosphate
cGMP: cyclic guanosine 3' 5'-monophosphate
PKA: cAMP-dependent protein kinase
PKG: cGMP-dependent protein kinase
PDE: phosphodiesterase
LFC: Log2FoldChange.

## References

[1] Bozkurt B Coats AJS Tsutsui H Abdelhamid CM Adamopoulos S Albert N. Universal definition and classification of heart failure: a report of the heart failure society of America, heart failure association of the European society of cardiology, japanese heart failure society and writing committee of the universal definition of heart failure: endorsed by the canadian heart failure society, heart failure association of India, cardiac society of Australia and New Zealand, and Chinese heart failure association. Eur J Heart Fail. (2021) 23:352–80. 10.1002/ejhf.2115 33605000

[2] Lorenz K Stathopoulou K Schmid E Eder P Cuello F. Heart failure-specific changes in protein kinase signalling. Pflügers Archiv Eur J Physiol. (2014) 466:1151. 10.1007/s00424-014-1462-x 24510065

[3] Fuller SJ Osborne SA Leonard SJ Hardyman MA Vaniotis G Allen BG. Cardiac protein kinases: the cardiomyocyte kinome and differential kinase expression in human failing hearts. Cardiovasc Res. (2015) 108:87–98. 10.1093/cvr/cvv210 26260799

[4] Guo Y Sui JY Kim K Zhang Z Qu XA Nam YJ. Cardiomyocyte homeodomain-interacting protein kinase 2 maintains basal cardiac function via extracellular signal-regulated kinase signaling. Circulation. (2019) 140:1820–33. 10.1161/CIRCULATIONAHA.119.040740 31581792 PMC7219521

[5] Hasin Y Seldin M Lusis A. Multi-omics approaches to disease. Genome Biol. (2017) 18:83. 10.1186/s13059-017-1215-1 28476144 PMC5418815

[6] Leon-Mimila P Wang J Huertas-Vazquez A. Relevance of multi-omics studies in cardiovascular diseases. Front Cardiovasc Med. (2019) 6:91. 10.3389/fcvm.2019.00091 31380393 PMC6656333

[7] Joshi A Rienks M Theofilatos K Mayr M. Systems biology in cardiovascular disease: a multiomics approach. Nat Rev Cardiol. (2021) 18:313–30. 10.1038/s41569-020-00477-1 33340009

[8] Ewels P Magnusson M Lundin S Käller M. MultiQC: summarize analysis results for multiple tools and samples in a single report. Bioinformatics. (2016) 32:3047–8. 10.1093/bioinformatics/btw354 27312411 PMC5039924

[9] Bolger AM Lohse M Usadel B. Trimmomatic: a flexible trimmer for illumina sequence data. Bioinformatics. (2014) 30:2114–20. 10.1093/bioinformatics/btu170 24695404 PMC4103590

[10] Dobin A Davis CA Schlesinger F Drenkow J Zaleski C Jha S. STAR: ultrafast universal RNA-seq aligner. Bioinformatics. (2013) 29:15–21. 10.1093/bioinformatics/bts635 23104886 PMC3530905

[11] Okonechnikov K Conesa A García-Alcalde F. Qualimap 2: advanced multi-sample quality control for high-throughput sequencing data. Bioinformatics. (2016) 32:292–4. 10.1093/bioinformatics/btv566 26428292 PMC4708105

[12] Liao Y Smyth GK Shi W. Featurecounts: an efficient general purpose program for assigning sequence reads to genomic features. Bioinformatics. (2014) 30:923–30. 10.1093/bioinformatics/btt656 24227677

[13] Love MI Huber W Anders S. Moderated estimation of fold change and dispersion for RNA-seq data with DESeq2. Genome Biol. (2014) 15:550. 10.1186/s13059-014-0550-8 25516281 PMC4302049

[14] Yates A Akanni W Amode MR Barrell D Billis K Carvalho-Silva D. Ensembl 2016. Nucleic Acids Res. (2016) 44:D710–6. 10.1093/nar/gkv1157 26687719 PMC4702834

[15] Huber W Durinck S Spellman PT Birney E. Mapping identifiers for the integration of genomic datasets with the R/Bioconductor package biomaRt. Nat Protoc. (2009) 4:1184–91. 10.1038/nprot.2009.97 19617889 PMC3159387

[16] Cox J Mann M. MaxQuant enables high peptide identification rates, individualized ppb-range mass accuracies and proteome-wide protein quantification. Nat Biotechnol. (2008) 26:1367–72. 10.1038/nbt.1511 19029910

[17] Cox J Neuhauser N Michalski A Scheltema RA Olsen Jv Mann M. Andromeda: a peptide search engine integrated into the maxquant environment. J Proteome Res. (2011) 10:1794–805. 10.1021/pr101065j 21254760

[18] Zhang X Smits AH van Tilburg GBA Ovaa H Huber W Vermeulen M. Proteome-wide identification of ubiquitin interactions using UbIA-MS. Nat Protoc. (2018) 13:530–50. 10.1038/nprot.2017.147 29446774

[19] Yu G Wang LG Han Y He QY. clusterProfiler: an R package for comparing biological themes among gene clusters. OMICS. (2012) 16:284–7. 10.1089/omi.2011.0118 22455463 PMC3339379

[20] Csárdi G Nepusz T. The igraph software package for complex network research. Int J Complex Syst. (2006) 1695:1–9.31819800

[21] Shannon P Markiel A Ozier O Baliga NS Wang JT Ramage D. Cytoscape: a software environment for integrated models of biomolecular interaction networks. Genome Res. (2003) 13:2498–504. 10.1101/gr.1239303 14597658 PMC403769

[22] Lang RM Badano LP Mor-Avi V Afilalo J Armstrong A Ernande L. Recommendations for cardiac chamber quantification by echocardiography in adults: an update from the american society of echocardiography and the european association of cardiovascular imaging. J Am Soc Echocardiogr. (2015) 28:1–39. 10.1016/j.echo.2014.10.003 25559473

[23] Brunner-La Rocca H Sanders - van Wijk S. Natriuretic peptides in chronic heart failure. Card Fail Rev. (2019) 5:44–9. 10.15420/cfr.2018.26.1 30847245 PMC6396059

[24] Felker GM Petersen JW Mark DB. Natriuretic peptides in the diagnosis and management of heart failure. Can Med Assoc J. (2006) 175:611–7. 10.1503/cmaj.060236 16966666 PMC1559415

[25] Alessi DR Pearce LR Komander D. The nuts and bolts of AGC protein kinases. Nat Rev Mol Cell Biol. (2010) 11:9–22. 10.1038/nrm2822 20027184

[26] Cheng X Ma Y Moore M Hemmings BA Taylor SS. Phosphorylation and activation of cAMP-dependent protein kinase by phosphoinositide-dependent protein kinase. Proc Natl Acad Sci PNAS. (1998) 95:9849–54. 10.1073/pnas.95.17.9849 9707564 PMC21425

[27] Joseph P Swedberg K Leong DP Yusuf S. The evolution of β-Blockers in coronary artery disease and heart failure (Part 1/5). J Am Coll Cardiol. (2019) 74:672–82. 10.1016/j.jacc.2019.04.067 31370960

[28] Colombe AS Pidoux G. Cardiac cAMP-PKA signaling compartmentalization in myocardial infarction. Cells. (2021) 10:922. 10.3390/cells10040922 33923648 PMC8073060

[29] Zimmermann B Chiorini JA Ma Y Kotin RM Herberg FW. PrKX is a novel catalytic subunit of the cAMP-dependent protein kinase regulated by the regulatory subunit type I. J Biol Chem. (1999) 274:5370–8. 10.1074/jbc.274.9.5370 10026146

[30] Li X Iomini C Hyink D Wilson PD. PRKX critically regulates endothelial cell proliferation, migration, and vascular-like structure formation. Dev Biol. (2011) 356:475–85. 10.1016/j.ydbio.2011.05.673 21684272

[31] Kong Q Blanton RM. Protein kinase G I and heart failure: shifting focus from vascular unloading to direct myocardial antiremodeling effects. Circ Heart Fail. (2013) 6:1268–83. 10.1161/CIRCHEARTFAILURE.113.000575 24255056 PMC4213067

[32] Rivero-Vilches FJ de Frutos S Saura M Rodriguez-Puyol D Rodriguez-Puyol M. Ro-driguez-Puyol D. Differential relaxing responses to particulate or soluble guanylyl cyclase activation on endothelial cells: a mechanism dependent on PKG-I activation by NO/cGMP. Am J Physiol Cell Physiol. (2003) 285:891–8. 10.1152/ajpcell.00590.2002 12814915

[33] Moyes AJ Hobbs AJ. C-Type natriuretic peptide: a multifaceted paracrine regulator in the heart and vasculature. Int J Mol Sci. (2019) 20:2281. 10.3390/ijms20092281 31072047 PMC6539462

[34] Moyes AJ Chu SM Aubdool AA Dukinfield MS Margulies KB Bedi KC. C-type natriuretic peptide co-ordinates cardiac structure and function. Eur Heart J. (2020) 41:1006–20. 10.1093/eurheartj/ehz093 30903134 PMC7068173

[35] Emdin M Aimo A Castiglione V Vergaro G Georgiopoulos G Saccaro LF. Targeting cyclic guanosine monophosphate to treat heart failure; JACC review topic of the week. J Am Coll Cardiol. (2020) 76:1795–807. 10.1016/j.jacc.2020.08.031 33032741

[36] Ichiki T Schirger JA Huntley BK Brozovich Fv Maleszewski JJ Sandberg SM. Cardiac fibrosis in end-stage human heart failure and the cardiac natriuretic peptide guanylyl cyclase system: Regulation and therapeutic implications. J Mol Cell Cardiol. (2014) 75:199–205. 10.1016/j.yjmcc.2014.08.001 25117468 PMC4157955

[37] Tarazón E Roselló-Lletí E Ortega A Molina-Navarro MM Sánchez-Lázaro I Lago F. Differential gene expression of C-type natriuretic peptide and its related molecules in dilated and ischemic cardiomyopathy. a new option for the management of heart failure. Int J Cardiol. (2014) 174:e84–6. 10.1016/j.ijcard.2014.04.037 24809913

[38] Niederkofler EE Kiernan UA O'Rear J Menon S Saghir S Protter AA. Detection of endogenous B-Type natriuretic peptide at very low concentrations in patients with heart failure. Circ Heart Fail. (2008) 1:258–64. 10.1161/CIRCHEARTFAILURE.108.790774 19808300

[39] Miller WL Phelps MA Wood CM Schellenberger U van Le A Perichon R. Comparison of mass spectrometry and clinical assay measurements of circulating fragments of B-type natriuretic peptide in patients with chronic heart failure. Circ Heart Fail. (2011) (3):355–60. 10.1161/CIRCHEARTFAILURE.110.960260 21292992

[40] Ibebuogu UN Gladysheva IP Houng AK Reed GL. Decompensated heart failure is associated with reduced corin levels and decreased cleavage of pro-atrial natriuretic peptide. Circ Heart Fail. (2011) 4:114–20. 10.1161/CIRCHEARTFAILURE.109.895581 21216831 PMC3840730

[41] Gommans DHF Revuelta-Lopez E Lupon J Cserkóová A Domingo M Vart P. Soluble neprilysin and corin concentrations in relation to clinical outcome in chronic heart failure. JACC Heart Fail. (2021) 9:85–95. 10.1016/j.jchf.2020.08.015 33189629

[42] Preedy MEJ. Cardiac cyclic nucleotide phosphodiesterases: roles and therapeutic potential in heart failure. Cardiovasc Drugs Ther. (2020) 34:401–17. 10.1007/s10557-020-06959-1 32172427 PMC7242274

[43] Zhang H Pan B Wu P Parajuli N Rekhter MD Goldberg AL. PDE1 inhibition facilitates proteasomal degradation of misfolded proteins and protects against cardiac proteinopathy. Sci Adv. (2019) 5:1–16. 10.1126/sciadv.aaw5870 31131329 PMC6531002

[44] Hashimoto T Kim G Tunin R Adesiyun T Hsu S Nakagawa R. Acute enhancement of cardiac function by phosphodiesterase type 1 inhibition: translational study in the dog and rabbit. Circulation. (2018) 138:1974–87. 10.1161/CIRCULATIONAHA.117.030490 30030415 PMC6205901

[45] Zhang Y Knight W Chen S Mohan A Yan C. Multiprotein complex with TRPC (Transient Receptor Potential-Canonical) channel, PDE1C (Phosphodiesterase 1C), and A2R (Adenosine A2 Receptor) plays a critical role in regulating cardiomyocyte camp and survival. Circulation. (2018) 138:1988–2002. 10.1161/CIRCULATIONAHA.118.034189 29871977 PMC6205915

[46] Meier S Andressen KW Aronsen JM Sjaastad I Hougen K Skomedal T. PDE3 inhibition by C-type natriuretic peptide-induced cGMP enhances cAMP-mediated signaling in both non-failing and failing hearts. Eur J Pharmacol. (2017) 812:174–83. 10.1016/j.ejphar.2017.07.014 28697992

[47] Rao MS van Vleet TR Ciurlionis R Buck WR Mittelstadt SW Blomme EAG. Comparison of RNA-Seq and microarray gene expression platforms for the toxicogenomic evaluation of liver from short-term rat toxicity studies. Front Genet. (2019) 9:636. 10.3389/fgene.2018.00636 30723492 PMC6349826

