# Supplementary material for: Alterations of Cardiac Protein Kinases in Cyclic Nucleotide-Dependent Signaling Pathways in Human Ischemic Heart Failure
Source: Front Cardiovasc Med. 2022 Jun 17;9:919355. doi: 10.3389/fcvm.2022.919355 (PMC9247256; doi:10.3389/fcvm.2022.919355)
Supplement: Supplementary file 1 [file Data_Sheet_1.DOCX]

**Supplementary Table 1. Primary antibody used in Western Blotting**

| Antibody | Dilution | Host | Clonality | Product number | Manufacturer |
| --- | --- | --- | --- | --- | --- |
| PKA-Cα | 1:3000 | Rabbit | Polyclonal | 4782S | Cell Signaling Technology |
| Phospho-PKA-Cα (Thr197) | 1:3000 | Rabbit | Polyclonal | 4781S | Cell Signaling Technology |
| PKG | 1:1000 | Rabbit | Polyclonal | MBS840764 | MyBioSource |
| Phospho-VASP (Ser239) | 1:1500 | Rabbit | Polyclonal | 3114S | Cell Signaling Technology |
| Ryanodine Receptor Monoclonal antibody (C3-33) | 1:1000 | Mouse | Monoclonal | MA3-916 | ThermoFisher scientific |
| Anti-Ryanodine receptor 2/RyR2 (phospho S2808) | 1:1000 | Rabbit | Polyclonal | ab59225 | Abcam |
| Kv1.1 (KCNA1) Recombinant Rabbit Monoclonal Antibody (SN66-06) | 1:3500 | Rabbit | Monoclonal | MA5-32317 | ThermoFisher scientific |
| Phospho-Kv1.1 (KCNA1) (Ser446) | 1:10000 | Rabbit | Polyclonal | PA5-105318 | ThermoFisher scientific |
| Anti-PRKX antibody [EPR4840] | 1:5000 | Rabbit | Monoclonal | ab109389 | Abcam |

**Supplementary Table 2. Significantly differentially phosphorylated STK and PTK peptide sequences**

| \| **STK ID** \| **Uniprot**  **Accession** \| **Sequence** \| **LFC^a^** \| **p value** \| \| --- \| --- \| --- \| --- \| --- \| \| RBL2_655_667 \| Q08999 \| GLGRSITSPTTLY \| 0.78 \| 0.00002 \| \| GPR6_349_361 \| P46095 \| QSKVPFRSRSPSE \| 1.05 \| 0.00004 \| \| ANDR_785_797 \| P10275 \| VRMRHLSQEFGWL \| 0.87 \| 0.00006 \| \| RAP1B_172_184 \| P61224 \| PGKARKKSSCQLL \| 0.72 \| 0.00007 \| \| BAD_69_81 \| Q92934 \| IRSRHSSYPAGTE \| 0.69 \| 0.00012 \| \| KIF2C_105_118_S106G \| Q99661 \| EGLRSRSTRMSTVS \| 0.69 \| 0.00012 \| \| KCNA2_442_454 \| P16389 \| PDLKKSRSASTIS \| 1.09 \| 0.00016 \| \| FRAP_2443_2455 \| P42345 \| RTRTDSYSAGQSV \| 1.21 \| 0.00020 \| \| BCKD_45_57 \| O14874 \| ERSKTVTSFYNQS \| 0.82 \| 0.00033 \| \| MPIP1_172_184 \| P30304 \| FTQRQNSAPARML \| 0.76 \| 0.00035 \| \| CAC1C_1974_1986 \| Q13936 \| ASLGRRASFHLEC \| 0.62 \| 0.00037 \| \| KCNA3_461_473 \| P22001 \| EELRKARSNSTLS \| 1.44 \| 0.00037 \| \| F263_454_466 \| Q16875 \| NPLMRRNSVTPLA \| 0.56 \| 0.00039 \| \| STK6_283_295 \| O14965 \| SSRRTTLCGTLDY \| 0.58 \| 0.00049 \| \| VASP_150_162 \| P50552 \| EHIERRVSNAGGP \| 1.04 \| 0.00053 \| \| GBRB2_427_439 \| P47870 \| SRLRRRASQLKIT \| 0.55 \| 0.00057 \| \| NOS3_1171_1183 \| P29474 \| SRIRTQSFSLQER \| 1.06 \| 0.00058 \| \| E1A_ADE05_212_224 \| P03255 \| AILRRPTSPVSRE \| 0.73 \| 0.00059 \| \| TOP2A_1463_1475 \| P11388 \| RRKRKPSTSDDSD \| 0.57 \| 0.00060 \| \| EPB42_241_253 \| P16452 \| LLNKRRGSVPILR \| 1.07 \| 0.00062 \| \| DESP_2842_2854 \| P15924 \| RSGSRRGSFDATG \| 1.54 \| 0.00067 \| \| STMN2_90_102 \| Q93045 \| AAGERRKSQEAQV \| 1.39 \| 0.00068 \| \| NMDZ1_890_902 \| Q05586 \| SFKRRRSSKDTST \| 0.90 \| 0.00071 \| \| ADRB2_338_350 \| P07550 \| ELLCLRRSSLKAY \| 0.72 \| 0.00072 \| \| ANXA1_209_221 \| P04083 \| AGERRKGTDVNVF \| 0.87 \| 0.00073 \| \| KAP2_92_104 \| P13861 \| SRFNRRVSVCAET \| 0.64 \| 0.00075 \| \| GRIK2_708_720 \| Q13002 \| FMSSRRQSVLVKS \| 0.58 \| 0.00078 \| \| GPSM2_394_406 \| P81274 \| PKLGRRHSMENME \| 1.37 \| 0.00078 \| \| CGHB_109_121 \| P01233 \| QCALCRRSTTDCG \| 0.68 \| 0.00087 \| \| BAD_112_124 \| Q92934 \| RELRRMSDEFVDS \| 1.73 \| 0.00089 \| \| CFTR_761_773 \| P13569 \| LQARRRQSVLNLM \| 0.42 \| 0.00090 \| \| PTN12_32_44 \| Q05209 \| FMRLRRLSTKYRT \| 0.57 \| 0.00100 \| \| KAP3_107_119 \| P31323 \| NRFTRRASVCAEA \| 0.45 \| 0.00100 \| \| REL_260_272 \| Q04864 \| KMQLRRPSDQEVS \| 1.16 \| 0.00117 \| \| CSF1R_701_713 \| P07333 \| NIHLEKKYVRRDS \| 0.95 \| 0.00121 \| \| PTK6_436_448 \| Q13882 \| ALRERLSSFTSYE \| 0.74 \| 0.00121 \| \| ESR1_160_172 \| P03372 \| GGRERLASTNDKG \| 0.58 \| 0.00138 \| \| NCF1_321_333 \| P14598 \| QDAYRRNSVRFLQ \| 0.79 \| 0.00139 \| \| ART_025_CXGLRRWSLGGLRRWSL \| Na \| GLRRWSLGGLRRWSL \| 0.89 \| 0.00155 \| \| NFKB1_330_342 \| P19838 \| FVQLRRKSDLETS \| 0.86 \| 0.00162 \| \| TY3H_65_77 \| P07101 \| FIGRRQSLIEDAR \| 0.67 \| 0.00163 \| \| KPB1_1011_1023 \| P46020 \| QVEFRRLSISAES \| 0.92 \| 0.00164 \| \| MYPC3_268_280 \| Q14896 \| LSAFRRTSLAGGG \| 0.64 \| 0.00164 \| \| PLM_76_88 \| O00168 \| EEGTFRSSIRRLS \| 1.31 \| 0.00170 \| \| RYR1_4317_4329 \| P21817 \| VRRLRRLTAREAA \| 0.62 \| 0.00182 \| \| KCNA1_438_450 \| Q09470 \| DSDLSRRSSSTMS \| 1.28 \| 0.00184 \| \| CFTR_730_742 \| P13569 \| EPLERRLSLVPDS \| 0.82 \| 0.00202 \| \| CREB1_126_138 \| P16220 \| EILSRRPSYRKIL \| 0.60 \| 0.00210 \| \| SCN7A_898_910 \| Q01118 \| KNGCRRGSSLGQI \| 0.95 \| 0.00212 \| \| VASP_271_283 \| P50552 \| LARRRKATQVGEK \| 0.65 \| 0.00255 \| \| LIPS_944_956 \| Q05469 \| GFHPRRSSQGATQ \| 0.97 \| 0.00266 \| \| KCNA6_504_516 \| P17658 \| ANRERRPSYLPTP \| 0.49 \| 0.00332 \| \| CDN1A_139_151 \| P38936 \| GRKRRQTSMTDFY \| 0.39 \| 0.00362 \| \| RS6_228_240 \| P62753 \| IAKRRRLSSLRAS \| 0.70 \| 0.00365 \| \| PPR1A_28_40 \| Q13522 \| QIRRRRPTPATLV \| 0.37 \| 0.00387 \| \| CENPA_1_14 \| P49450 \| MGPRRRSRKPEAPR \| 0.38 \| 0.00567 \| \| RAF1_253_265 \| P04049 \| QRQRSTSTPNVHM \| 0.62 \| 0.00695 \| \| VTNC_390_402 \| P04004 \| NQNSRRPSRATWL \| 0.65 \| 0.00762 \| \| MPIP3_208_220 \| P30307 \| RSGLYRSPSMPEN \| 0.72 \| 0.00921 \| \| KPCB_19_31_A25S \| P05771 \| RFARKGSLRQKNV \| 0.53 \| 0.01088 \| \| NCF1_296_308 \| P14598 \| RGAPPRRSSIRNA \| 0.65 \| 0.01169 \| \| PLEK_106_118 \| P08567 \| GQKFARKSTRRSI \| 0.28 \| 0.02677 \| \| CA2D1_494_506 \| P54289 \| LEDIKRLTPRFTL \| 0.36 \| 0.03070 \| \|  \|  \|  \|  \|  \| \|  \|  \|  \|  \|  \| \| **PTK ID** \| **Uniprot**  **Accession** \| **Sequence** \| **LFC^a^** \| **p value** \| \| JAK1_1027_1039 \| P23458 \| AIETDKEYYTVKD \| 0.96 \| 0.00339 \| \| JAK2_563_577 \| O60674 \| VRREVGDYGQLHETE \| 1.13 \| 0.00355 \| \| ART_004_EAIYAAPFAKKKXC \| Na \| EAIYAAPFAKKK \| 0.66 \| 0.00467 \| \| CDK4_11_23 \| P11802 \| EIGVGAYGTVYKA \| 1.27 \| 0.00616 \| \| LAT_194_206 \| O43561 \| MESIDDYVNVPES \| 2.02 \| 0.00827 \| \| MAPK3_198_210_C203S \| Q16644 \| ALQTPSYTPYYVA \| 1.93 \| 0.01029 \| \| KSYK_518_530 \| P43405 \| ALRADENYYKAQT \| 1.04 \| 0.01136 \| \| FGFR3_753_765 \| P22607 \| TVTSTDEYLDLSA \| 1.69 \| 0.01236 \| \| PGFRB_1014_1028 \| P09619 \| PNEGDNDYIIPLPDP \| 2.18 \| 0.01274 \| \| ANXA1_14_26 \| P04083 \| IENEEQEYVQTVK \| 1.21 \| 0.01297 \| \| CBL_693_705 \| P22681 \| EGEEDTEYMTPSS \| 2.49 \| 0.01354 \| \| EPHA7_607_619 \| Q15375 \| TYIDPETYEDPNR \| 0.65 \| 0.01412 \| \| LAT_249_261 \| O43561 \| EEGAPDYENLQEL \| 0.84 \| 0.01803 \| \| EPOR_361_373 \| P19235 \| SEHAQDTYLVLDK \| 0.76 \| 0.01966 \| \| PDPK1_369_381 \| O15530 \| DEDCYGNYDNLLS \| 0.47 \| 0.01993 \| \| FGFR2_762_774 \| P21802 \| TLTTNEEYLDLSQ \| 0.69 \| 0.02047 \| \| ERBB2_1241_1253 \| P04626 \| PTAENPEYLGLDV \| 0.85 \| 0.02053 \| \| ERBB2_870_882 \| P04626 \| LDIDETEYHADGG \| 1.28 \| 0.02090 \| \| LYN_391_403 \| P07948 \| VIEDNEYTAREGA \| 1.29 \| 0.02973 \| \| PGFRB_768_780 \| P09619 \| SSNYMAPYDNYVP \| 1.10 \| 0.03314 \| \| EGFR_1165_1177 \| P00533 \| ISLDNPDYQQDFF \| 0.33 \| 0.03327 \| \| JAK3_974_986 \| P52333 \| LPLDKDYYVVREP \| 0.47 \| 0.03494 \| \| MK03_199_208 \| P27361 \| GFLTEYVATR \| 1.07 \| 0.03892 \| \| BTLA_252_262 \| Q7Z6A9 \| KPGIVYASLNH \| 1.80 \| 0.03899 \| \| EPHB1_771_783 \| P54762 \| DDTSDPTYTSSLG \| 0.62 \| 0.04300 \| \| AKT1_309_321_C310S \| P31749 \| FSGTPEYLAPEVL \| 0.86 \| 0.04390 \| \| PP2AB_297_309 \| P62714 \| EPHVTRRTPDYFL \| 0.97 \| 0.04412 \| \| PTN11_57_67 \| Q06124 \| QNTGDYYDLYG \| 0.40 \| 0.04699 \| |
| --- | --- | --- | --- | --- | --- | --- | --- | --- | --- | --- | --- | --- | --- | --- | --- | --- | --- | --- | --- | --- | --- | --- | --- | --- | --- | --- | --- | --- | --- | --- | --- | --- | --- | --- | --- | --- | --- | --- | --- | --- | --- | --- | --- | --- | --- | --- | --- | --- | --- | --- | --- | --- | --- | --- | --- | --- | --- | --- | --- | --- | --- | --- | --- | --- | --- | --- | --- | --- | --- | --- | --- | --- | --- | --- | --- | --- | --- | --- | --- | --- | --- | --- | --- | --- | --- | --- | --- | --- | --- | --- | --- | --- | --- | --- | --- | --- | --- | --- | --- | --- | --- | --- | --- | --- | --- | --- | --- | --- | --- | --- | --- | --- | --- | --- | --- | --- | --- | --- | --- | --- | --- | --- | --- | --- | --- | --- | --- | --- | --- | --- | --- | --- | --- | --- | --- | --- | --- | --- | --- | --- | --- | --- | --- | --- | --- | --- | --- | --- | --- | --- | --- | --- | --- | --- | --- | --- | --- | --- | --- | --- | --- | --- | --- | --- | --- | --- | --- | --- | --- | --- | --- | --- | --- | --- | --- | --- | --- | --- | --- | --- | --- | --- | --- | --- | --- | --- | --- | --- | --- | --- | --- | --- | --- | --- | --- | --- | --- | --- | --- | --- | --- | --- | --- | --- | --- | --- | --- | --- | --- | --- | --- | --- | --- | --- | --- | --- | --- | --- | --- | --- | --- | --- | --- | --- | --- | --- | --- | --- | --- | --- | --- | --- | --- | --- | --- | --- | --- | --- | --- | --- | --- | --- | --- | --- | --- | --- | --- | --- | --- | --- | --- | --- | --- | --- | --- | --- | --- | --- | --- | --- | --- | --- | --- | --- | --- | --- | --- | --- | --- | --- | --- | --- | --- | --- | --- | --- | --- | --- | --- | --- | --- | --- | --- | --- | --- | --- | --- | --- | --- | --- | --- | --- | --- | --- | --- | --- | --- | --- | --- | --- | --- | --- | --- | --- | --- | --- | --- | --- | --- | --- | --- | --- | --- | --- | --- | --- | --- | --- | --- | --- | --- | --- | --- | --- | --- | --- | --- | --- | --- | --- | --- | --- | --- | --- | --- | --- | --- | --- | --- | --- | --- | --- | --- | --- | --- | --- | --- | --- | --- | --- | --- | --- | --- | --- | --- | --- | --- | --- | --- | --- | --- | --- | --- | --- | --- | --- | --- | --- | --- | --- | --- | --- | --- | --- | --- | --- | --- | --- | --- | --- | --- | --- | --- | --- | --- | --- | --- | --- | --- | --- | --- | --- | --- | --- | --- | --- | --- | --- | --- | --- | --- | --- | --- | --- | --- | --- | --- | --- | --- | --- | --- | --- | --- | --- | --- | --- | --- | --- | --- | --- | --- | --- | --- | --- | --- | --- | --- | --- | --- | --- | --- | --- | --- | --- | --- | --- | --- | --- | --- | --- | --- | --- | --- | --- | --- | --- | --- | --- | --- | --- | --- | --- | --- | --- | --- | --- | --- | --- | --- | --- | --- | --- | --- | --- | --- | --- | --- | --- | --- | --- | --- | --- | --- | --- | --- |

^a^Log2FoldChange

**Supplementary Table 3. Top predicted STK and PTK**

| Rank | STK | Median final score^a^ | Mean kinase statistic^b^ | Rank | PTK | Median final score | Mean kinase statistic |
| --- | --- | --- | --- | --- | --- | --- | --- |
| 1 | PKA[alpha] | 5.40 | 1.42 | 1 | BLK | 3.42 | 0.67 |
| 2 | AMPK[alpha]1 | 5.40 | 1.72 | 2 | Fes | 2.78 | 0.64 |
| 3 | PKG1 | 5.40 | 1.47 | 3 | Yes | 2.77 | 0.65 |
| 4 | PKG2 | 5.10 | 1.47 | 4 | Brk | 2.57 | 0.59 |
| 5 | p70S6K[beta] | 4.62 | 1.50 | 5 | PDGFR[beta] | 2.27 | 0.62 |
| 6 | Akt1/PKB[alpha] | 4.28 | 1.44 | 6 | TEC | 2.15 | 0.59 |
| 7 | PRKX | 4.25 | 1.42 | 7 | Lck | 2.13 | 0.60 |
| 8 | CHK2 | 3.79 | 1.43 | 8 | Src | 2.13 | 0.57 |
| 9 | MAPKAPK2 | 3.68 | 1.39 | 9 | FGFR1 | 2.12 | 0.63 |
| 10 | PKD1 | 3.68 | 1.57 | 10 | Ret | 2.07 | 0.58 |
| 11 | CK2[alpha]1 | 3.61 | 1.67 | 11 | ZAP70 | 2.07 | 0.57 |
| 12 | CK1[epsilon] | 3.61 | 1.69 | 12 | FRK | 2.02 | 0.56 |
| 13 | IKK[alpha] | 3.51 | 1.62 | 13 | HER4 | 1.97 | 0.61 |
| 14 | Pim1 | 3.50 | 1.29 | 14 | Syk | 1.86 | 0.56 |
| 15 | MAPKAPK3 | 3.49 | 1.35 | 15 | Etk/BMX | 1.82 | 0.58 |
| 16 | Akt2/PKB[beta] | 3.49 | 1.36 | 16 | HER3 | 1.76 | 0.56 |
| 17 | CaMK4 | 3.42 | 1.42 | 17 | EphA2 | 1.74 | 0.62 |
| 18 | PRKY | 3.40 | 1.41 | 18 | HER2 | 1.71 | 0.57 |
| 19 | PFTAIRE1 | 3.39 | 1.59 | 19 | FLT4 | 1.71 | 0.59 |
| 20 | RSK1/p90RSK | 3.39 | 1.46 | 20 | Met | 1.67 | 0.55 |

^a^Median final score: used for ranking kinases based on their significance and specificity in terms of the set of peptides used for the corresponding kinase.

^b^Mean kinase statistic: indicates the overall change of the peptide set that represents the kinase.

**Supplementary Table 4. Differential gene expression of the top STKs and PTKs**

| STKs  Gene name | Description | LFC^a^ | padj^b^ |
| --- | --- | --- | --- |
| *PRKAA1* | Protein kinase AMP-activated catalytic subunit alpha 1, AMPK alpha 1 | 0.04 | n.s. |
| *RPS6KB1* | Ribosomal protein S6 kinase B1, P70S6K1 | -0.09 | n.s. |
| *AKT1* | AKT serine/threonine kinase 1, Protein kinase B alpha,. PKB | -0.20 | n.s. |
| *CHEK2* | Checkpoint kinase 2 | -0.56 | n.s. |
| *MAPKAPK2* | MAPK activated protein kinase 2 | -0.42 | 4.1E-04 *** |
| *PKD1* | Polycystin 1, transient receptor potential channel interacting | 0.18 | n.s. |
| *CSNK2A1* | Casein kinase 2 alpha 1 | -0.03 | n.s. |
| *CSNK1E* | Casein kinase 1 epsilon | -0.03 | n.s. |
| *CHUK* | Component of inhibitor of nuclear factor kappa B kinase complex | -0.10 | n.s. |
| *PIM1* | Pim-1 proto-oncogene, serine/threonine kinase | -1.04 | 0.054 |
| *MAPKAPK3* | MAPK activated protein kinase 3 | -0.54 | 0.02 * |
| *AKT2* | AKT serine/threonine kinase 2, Protein kinase B beta | -0.19 | 0.002 ** |
| *CAMK4* | Calcium/calmodulin dependent protein kinase IV | 0.15 | n.s. |
| *PRKY* | protein kinase Y-linked (pseudogene) | -0.55 | n.s. |
| *PFTAIRE1* | Cyclin dependent kinase 14 | 0.02 | n.s. |
| *RPS6KA1* | Ribosomal protein S6 kinase A1 | -0.42 | n.s. |
| PTKs  Gene name | **Description** | **LFC^a^** | **padj^b^** |
| *BLK* | BLK proto-oncogene, Src family tyrosine kinase | 0.22 | n/a |
| *FES* | FES proto-oncogene, tyrosine kinase | -0.15 | n.s. |
| *YES1* | YES proto-oncogene 1, Src family tyrosine kinase | -0.21 | n.s. |
| *PTK6* | Protein tyrosine kinase 6, BRK | -0.06 | n/a |
| *PDGFRB* | Platelet derived growth factor receptor beta | -0.68 | 4.2E-04 *** |
| *TEC* | TEC protein tyrosine kinase | 0.05 | n.s. |
| *LCK* | LCK proto-oncogene, Src family tyrosine kinase | -0.59 | n.s. |
| *SRC* | SRC proto-oncogene, non-receptor tyrosine kinase | -0.37 | n.s. |
| *FGFR1* | Fibroblast growth factor receptor 1 | 0.20 | n.s. |
| *RET* | Ret proto-oncogene | -0.27 | n.s. |
| *ZAP70* | Zeta chain of T cell receptor associated protein kinase 70 | -0.09 | n.s. |
| *FRK* | Fyn related Src family tyrosine kinase | -0.12 | n.s. |
| *ERBB4* | Erb-b2 receptor tyrosine kinase 4, HER4 | 0.13 | n.s. |
| *SYK* | Spleen associated tyrosine kinase | -0.01 | n.s. |
| *BMX* | BMX non-receptor tyrosine kinase | 1.18 | 0.008 ** |
| *ERBB3* | Erb-b2 receptor tyrosine kinase 3, HER3 | -0.78 | n.s. |
| *EPHA2* | EPH receptor A2 | -0.12 | n.s. |
| *ERBB2* | Erb-b2 receptor tyrosine kinase 2 | -0.40 | 0.06 |
| *FLT4* | Fms related tyrosine kinase 4 | 0.08 | n.s. |
| *MET* | MET proto-oncogene,. receptor tyrosine kinase | -0.09 | n.s. |

^a^Log2FoldChange

^b^Adjusted p values less than 0.05 are regarded as statistically significant. * padj < 0.05, **

padj < 0.01, *** padj < 0.001.

^c^n.s. not significant

^d^n/a not available

**Supplementary Table 5. Significantly up-regulated protein coding mRNAs based on padj**

| **Gene_id** | **Gene_name** | **LFC^a^** | **p value** | **padj** |
| --- | --- | --- | --- | --- |
| ENSG00000100276.10 | RASL10A | 2.41 | 5.39E-15 | 7.77E-12 |
| ENSG00000158445.10 | KCNB1 | 2.54 | 1.71E-14 | 1.99E-11 |
| ENSG00000158816.15 | VWA5B1 | 4.17 | 2.08E-12 | 1.41E-09 |
| ENSG00000108551.5 | RASD1 | 2.96 | 3.80E-12 | 2.38E-09 |
| ENSG00000206190.12 | ATP10A | 2.13 | 6.56E-12 | 3.91E-09 |
| ENSG00000128965.13 | CHAC1 | 2.52 | 1.47E-11 | 7.49E-09 |
| ENSG00000179542.16 | SLITRK4 | 2.29 | 1.47E-11 | 7.49E-09 |
| ENSG00000168389.17 | MFSD2A | 2.20 | 1.91E-11 | 9.16E-09 |
| ENSG00000171747.9 | LGALS4 | 2.07 | 3.92E-11 | 1.75E-08 |
| ENSG00000271447.6 | MMP28 | 1.52 | 6.66E-11 | 2.55E-08 |
| ENSG00000147036.11 | LANCL3 | 2.64 | 1.04E-10 | 3.74E-08 |
| ENSG00000165449.11 | SLC16A9 | 2.18 | 2.13E-10 | 6.61E-08 |
| ENSG00000112214.10 | FHL5 | 1.55 | 6.58E-10 | 1.59E-07 |
| ENSG00000183134.5 | PTGDR2 | 1.92 | 1.03E-09 | 2.34E-07 |
| ENSG00000143127.13 | ITGA10 | 2.16 | 2.50E-09 | 5.11E-07 |
| ENSG00000118777.12 | ABCG2 | 1.75 | 2.94E-09 | 5.86E-07 |
| ENSG00000167723.14 | TRPV3 | 1.98 | 3.53E-09 | 6.70E-07 |
| ENSG00000166523.8 | CLEC4E | 2.39 | 1.67E-08 | 2.52E-06 |
| ENSG00000007314.12 | SCN4A | 2.47 | 2.44E-08 | 3.49E-06 |
| ENSG00000092068.20 | SLC7A8 | 1.62 | 3.07E-08 | 4.27E-06 |
| ENSG00000120937.9 | NPPB | 5.18 | 3.12E-08 | 4.27E-06 |
| ENSG00000118523.6 | CCN2 | 2.15 | 3.43E-08 | 4.54E-06 |
| ENSG00000164266.10 | SPINK1 | 3.36 | 3.53E-08 | 4.65E-06 |
| ENSG00000174938.14 | SEZ6L2 | 1.52 | 8.66E-08 | 9.81E-06 |
| ENSG00000179593.16 | ALOX15B | 2.31 | 8.65E-07 | 6.55E-05 |
| ENSG00000170703.15 | TTLL6 | 2.89 | 8.99E-07 | 6.71E-05 |
| ENSG00000112984.12 | KIF20A | 2.99 | 1.71E-06 | 1.10E-04 |
| ENSG00000173705.9 | SUSD5 | 2.29 | 1.70E-06 | 1.10E-04 |
| ENSG00000096060.14 | FKBP5 | 1.71 | 1.83E-06 | 1.16E-04 |
| ENSG00000144406.19 | UNC80 | 2.88 | 1.92E-06 | 1.20E-04 |
| ENSG00000145832.14 | SLC25A48 | 3.06 | 2.16E-06 | 1.33E-04 |
| ENSG00000101542.10 | CDH20 | 3.78 | 2.64E-06 | 1.54E-04 |
| ENSG00000056487.16 | PHF21B | 1.97 | 2.73E-06 | 1.58E-04 |
| ENSG00000106258.15 | CYP3A5 | 3.25 | 2.84E-06 | 1.63E-04 |
| ENSG00000196090.12 | PTPRT | 1.95 | 2.93E-06 | 1.67E-04 |
| ENSG00000049089.15 | COL9A2 | 1.84 | 3.21E-06 | 1.79E-04 |
| ENSG00000170579.17 | DLGAP1 | 1.72 | 4.01E-06 | 2.14E-04 |
| ENSG00000156265.16 | MAP3K7CL | 1.66 | 5.66E-06 | 2.84E-04 |
| ENSG00000214787.10 | MS4A4E | 1.67 | 8.71E-06 | 4.09E-04 |
| ENSG00000266524.3 | GDF10 | 2.18 | 1.10E-05 | 4.82E-04 |
| ENSG00000189431.8 | RASSF10 | 1.50 | 1.22E-05 | 5.23E-04 |
| ENSG00000125740.14 | FOSB | 3.96 | 1.47E-05 | 6.11E-04 |
| ENSG00000134042.13 | MRO | 1.74 | 1.49E-05 | 6.15E-04 |
| ENSG00000086717.18 | PPEF1 | 2.23 | 1.65E-05 | 6.71E-04 |
| ENSG00000004948.15 | CALCR | 4.46 | 1.70E-05 | 6.84E-04 |
| ENSG00000231274.5 | SBK3 | 1.51 | 1.70E-05 | 6.85E-04 |
| ENSG00000168356.12 | SCN11A | 1.76 | 1.75E-05 | 7.01E-04 |
| ENSG00000135480.16 | KRT7 | 3.48 | 2.06E-05 | 7.90E-04 |
| ENSG00000102924.12 | CBLN1 | 1.65 | 2.37E-05 | 8.75E-04 |
| ENSG00000179300.4 | RTL3 | 2.57 | 2.66E-05 | 9.47E-04 |
| ENSG00000089847.12 | ANKRD24 | 1.74 | 3.06E-05 | 1.06E-03 |
| ENSG00000100095.19 | SEZ6L | 2.50 | 3.23E-05 | 1.10E-03 |
| ENSG00000118432.12 | CNR1 | 2.02 | 4.29E-05 | 1.38E-03 |
| ENSG00000127324.9 | TSPAN8 | 2.29 | 4.34E-05 | 1.39E-03 |
| ENSG00000164270.17 | HTR4 | 1.95 | 4.95E-05 | 1.53E-03 |
| ENSG00000107859.10 | PITX3 | 1.81 | 5.81E-05 | 1.71E-03 |
| ENSG00000175206.10 | NPPA | 4.61 | 6.81E-05 | 1.93E-03 |
| ENSG00000127083.7 | OMD | 1.79 | 7.89E-05 | 2.16E-03 |
| ENSG00000279968.2 | GVQW2 | 1.57 | 9.28E-05 | 2.43E-03 |
| ENSG00000165730.16 | STOX1 | 1.75 | 9.80E-05 | 2.53E-03 |
| ENSG00000150394.14 | CDH8 | 1.98 | 1.20E-04 | 2.91E-03 |
| ENSG00000122025.15 | FLT3 | 1.51 | 1.27E-04 | 3.04E-03 |
| ENSG00000186583.12 | SPATC1 | 3.52 | 1.31E-04 | 3.10E-03 |
| ENSG00000175318.12 | GRAMD2A | 2.65 | 1.47E-04 | 3.37E-03 |
| ENSG00000116983.13 | HPCAL4 | 2.00 | 1.47E-04 | 3.37E-03 |
| ENSG00000165323.15 | FAT3 | 1.50 | 1.57E-04 | 3.56E-03 |
| ENSG00000130054.4 | FAM155B | 1.57 | 1.65E-04 | 3.70E-03 |
| ENSG00000178401.16 | DNAJC22 | 2.55 | 1.74E-04 | 3.84E-03 |
| ENSG00000166342.19 | NETO1 | 3.72 | 2.00E-04 | 4.24E-03 |
| ENSG00000106302.10 | HYAL4 | 2.09 | 2.01E-04 | 4.24E-03 |
| ENSG00000106366.9 | SERPINE1 | 2.04 | 2.42E-04 | 4.84E-03 |
| ENSG00000125966.10 | MMP24 | 1.74 | 2.52E-04 | 5.00E-03 |
| ENSG00000101938.15 | CHRDL1 | 1.85 | 2.83E-04 | 5.43E-03 |
| ENSG00000126562.17 | WNK4 | 2.01 | 2.97E-04 | 5.61E-03 |
| ENSG00000221986.7 | MYBPHL | 1.81 | 3.29E-04 | 6.09E-03 |
| ENSG00000196581.11 | AJAP1 | 1.71 | 3.35E-04 | 6.18E-03 |
| ENSG00000102468.10 | HTR2A | 3.11 | 3.37E-04 | 6.22E-03 |
| ENSG00000157343.8 | ARMC12 | 2.19 | 4.16E-04 | 7.34E-03 |
| ENSG00000137869.15 | CYP19A1 | 1.61 | 5.02E-04 | 8.48E-03 |
| ENSG00000173825.7 | TIGD3 | 1.51 | 5.23E-04 | 8.73E-03 |
| ENSG00000115665.9 | SLC5A7 | 1.92 | 5.30E-04 | 8.81E-03 |
| ENSG00000101306.11 | MYLK2 | 1.75 | 5.35E-04 | 8.86E-03 |
| ENSG00000106809.11 | OGN | 1.61 | 5.37E-04 | 8.87E-03 |
| ENSG00000106483.12 | SFRP4 | 2.53 | 5.55E-04 | 9.09E-03 |
| ENSG00000163576.18 | EFHB | 2.48 | 5.82E-04 | 9.43E-03 |
| ENSG00000132975.8 | GPR12 | 1.68 | 6.65E-04 | 1.04E-02 |
| ENSG00000112309.11 | B3GAT2 | 2.07 | 7.78E-04 | 1.17E-02 |
| ENSG00000188089.13 | PLA2G4E | 3.66 | 7.85E-04 | 1.18E-02 |
| ENSG00000173227.14 | SYT12 | 2.00 | 7.95E-04 | 1.19E-02 |
| ENSG00000125144.13 | MT1G | 2.57 | 8.58E-04 | 1.26E-02 |
| ENSG00000198570.5 | RD3 | 2.83 | 9.00E-04 | 1.30E-02 |
| ENSG00000133063.16 | CHIT1 | 2.27 | 1.09E-03 | 1.50E-02 |
| ENSG00000156466.10 | GDF6 | 1.94 | 1.11E-03 | 1.52E-02 |
| ENSG00000214866.9 | DCDC2C | 3.75 | 1.15E-03 | 1.56E-02 |
| ENSG00000157152.17 | SYN2 | 1.80 | 1.15E-03 | 1.56E-02 |
| ENSG00000004838.14 | ZMYND10 | 2.07 | 1.29E-03 | 1.69E-02 |
| ENSG00000004799.8 | PDK4 | 1.80 | 1.39E-03 | 1.79E-02 |
| ENSG00000135903.19 | PAX3 | 4.61 | 1.43E-03 | 1.82E-02 |
| ENSG00000168676.11 | KCTD19 | 3.01 | 1.59E-03 | 1.97E-02 |
| ENSG00000124343.13 | XG | 2.21 | 1.76E-03 | 2.14E-02 |
| ENSG00000053108.17 | FSTL4 | 3.40 | 1.83E-03 | 2.20E-02 |
| ENSG00000162998.5 | FRZB | 1.78 | 1.91E-03 | 2.26E-02 |
| ENSG00000198336.9 | MYL4 | 2.21 | 1.92E-03 | 2.27E-02 |
| ENSG00000197769.6 | MAP1LC3C | 1.66 | 1.93E-03 | 2.27E-02 |
| ENSG00000122375.12 | OPN4 | 1.50 | 1.93E-03 | 2.27E-02 |
| ENSG00000155265.11 | GOLGA7B | 1.55 | 2.01E-03 | 2.34E-02 |
| ENSG00000116176.6 | TPSG1 | 2.03 | 2.15E-03 | 2.46E-02 |
| ENSG00000164188.8 | RANBP3L | 1.53 | 2.25E-03 | 2.54E-02 |
| ENSG00000165966.16 | PDZRN4 | 1.89 | 2.28E-03 | 2.56E-02 |
| ENSG00000101098.12 | RIMS4 | 2.41 | 2.31E-03 | 2.59E-02 |
| ENSG00000272899.4 | ATP6V1FNB | 2.16 | 2.68E-03 | 2.87E-02 |
| ENSG00000179455.9 | MKRN3 | 2.65 | 2.78E-03 | 2.96E-02 |
| ENSG00000149506.11 | ZP1 | 1.79 | 3.02E-03 | 3.12E-02 |
| ENSG00000073737.16 | DHRS9 | 1.87 | 3.07E-03 | 3.15E-02 |
| ENSG00000062038.14 | CDH3 | 2.39 | 3.12E-03 | 3.18E-02 |
| ENSG00000225526.4 | MKRN2OS | 1.97 | 3.16E-03 | 3.21E-02 |
| ENSG00000104059.4 | FAM189A1 | 2.47 | 3.16E-03 | 3.21E-02 |
| ENSG00000133110.15 | POSTN | 1.80 | 3.27E-03 | 3.29E-02 |
| ENSG00000107984.10 | DKK1 | 1.78 | 3.27E-03 | 3.29E-02 |
| ENSG00000113763.12 | UNC5A | 2.19 | 3.34E-03 | 3.32E-02 |
| ENSG00000101251.12 | SEL1L2 | 1.72 | 3.42E-03 | 3.38E-02 |
| ENSG00000081277.12 | PKP1 | 1.80 | 4.04E-03 | 3.81E-02 |
| ENSG00000134343.14 | ANO3 | 2.13 | 4.06E-03 | 3.83E-02 |
| ENSG00000166866.13 | MYO1A | 1.67 | 4.08E-03 | 3.84E-02 |
| ENSG00000131771.14 | PPP1R1B | 1.75 | 4.62E-03 | 4.20E-02 |
| ENSG00000186564.5 | FOXD2 | 1.68 | 4.74E-03 | 4.27E-02 |
| ENSG00000143816.8 | WNT9A | 1.83 | 5.26E-03 | 4.58E-02 |
| ENSG00000188916.9 | INSYN2A | 1.68 | 5.28E-03 | 4.58E-02 |
| ENSG00000131044.17 | TTLL9 | 2.02 | 5.47E-03 | 4.68E-02 |

^a^Log2FoldChange, only the genes with LFC ≥ 1.5 are presented.

**Supplementary Table 6. Significantly down-regulated protein coding mRNAs based on padj**

| **Gene_id** | **Gene_name** | **LFC^a^** | **p value** | **padj** |
| --- | --- | --- | --- | --- |
| ENSG00000163762.7 | TM4SF18 | -1.69 | 1.31E-20 | 6.40E-17 |
| ENSG00000072310.17 | SREBF1 | -1.82 | 1.03E-19 | 4.19E-16 |
| ENSG00000118849.10 | RARRES1 | -5.06 | 1.56E-14 | 1.91E-11 |
| ENSG00000163273.4 | NPPC | -5.29 | 2.64E-14 | 2.93E-11 |
| ENSG00000248144.6 | ADH1C | -2.30 | 3.73E-14 | 3.81E-11 |
| ENSG00000166592.12 | RRAD | -1.98 | 4.14E-12 | 2.53E-09 |
| ENSG00000134817.10 | APLNR | -2.06 | 3.94E-11 | 1.75E-08 |
| ENSG00000196517.12 | SLC6A9 | -2.16 | 4.58E-11 | 1.93E-08 |
| ENSG00000183615.6 | FAM167B | -2.52 | 3.81E-10 | 1.07E-07 |
| ENSG00000160179.18 | ABCG1 | -1.55 | 7.77E-10 | 1.85E-07 |
| ENSG00000127129.10 | EDN2 | -3.05 | 1.07E-09 | 2.39E-07 |
| ENSG00000177374.13 | HIC1 | -1.70 | 1.23E-09 | 2.70E-07 |
| ENSG00000172156.4 | CCL11 | -3.12 | 2.44E-09 | 5.06E-07 |
| ENSG00000138944.8 | SHISAL1 | -2.20 | 2.65E-09 | 5.36E-07 |
| ENSG00000167680.16 | SEMA6B | -1.70 | 3.30E-09 | 6.45E-07 |
| ENSG00000152760.10 | TCTEX1D1 | -1.91 | 4.63E-09 | 8.45E-07 |
| ENSG00000100526.20 | CDKN3 | -1.72 | 5.07E-09 | 9.12E-07 |
| ENSG00000204655.12 | MOG | -2.36 | 1.15E-08 | 1.86E-06 |
| ENSG00000129173.13 | E2F8 | -1.63 | 1.21E-08 | 1.95E-06 |
| ENSG00000163435.16 | ELF3 | -3.62 | 3.11E-08 | 4.27E-06 |
| ENSG00000184261.5 | KCNK12 | -3.93 | 3.76E-08 | 4.90E-06 |
| ENSG00000135472.9 | FAIM2 | -1.96 | 8.41E-08 | 9.57E-06 |
| ENSG00000097046.13 | CDC7 | -2.20 | 1.09E-07 | 1.17E-05 |
| ENSG00000177363.5 | LRRN4CL | -1.57 | 1.38E-07 | 1.42E-05 |
| ENSG00000148344.11 | PTGES | -2.41 | 1.45E-07 | 1.48E-05 |
| ENSG00000122861.16 | PLAU | -1.84 | 2.04E-07 | 1.96E-05 |
| ENSG00000182272.12 | B4GALNT4 | -1.78 | 2.19E-07 | 2.08E-05 |
| ENSG00000175894.18 | TSPEAR | -3.06 | 3.75E-07 | 3.27E-05 |
| ENSG00000158486.13 | DNAH3 | -1.98 | 3.90E-07 | 3.39E-05 |
| ENSG00000137571.11 | SLCO5A1 | -1.75 | 7.39E-07 | 5.76E-05 |
| ENSG00000104848.1 | KCNA7 | -3.27 | 7.56E-07 | 5.85E-05 |
| ENSG00000196136.18 | SERPINA3 | -2.28 | 9.12E-07 | 6.74E-05 |
| ENSG00000116833.14 | NR5A2 | -1.60 | 1.01E-06 | 7.22E-05 |
| ENSG00000163220.11 | S100A9 | -2.22 | 1.85E-06 | 1.16E-04 |
| ENSG00000133874.2 | RNF122 | -1.64 | 1.95E-06 | 1.21E-04 |
| ENSG00000108691.9 | CCL2 | -3.14 | 2.18E-06 | 1.33E-04 |
| ENSG00000172935.9 | MRGPRF | -1.53 | 2.23E-06 | 1.35E-04 |
| ENSG00000105639.19 | JAK3 | -1.63 | 2.52E-06 | 1.47E-04 |
| ENSG00000230778.1 | ANKRD63 | -4.10 | 3.22E-06 | 1.79E-04 |
| ENSG00000160013.9 | PTGIR | -1.95 | 3.86E-06 | 2.07E-04 |
| ENSG00000123689.6 | G0S2 | -1.55 | 4.03E-06 | 2.15E-04 |
| ENSG00000133962.8 | CATSPERB | -1.85 | 4.48E-06 | 2.34E-04 |
| ENSG00000134107.5 | BHLHE40 | -1.96 | 4.60E-06 | 2.39E-04 |
| ENSG00000156966.7 | B3GNT7 | -2.55 | 1.23E-05 | 5.27E-04 |
| ENSG00000140285.10 | FGF7 | -1.62 | 1.33E-05 | 5.62E-04 |
| ENSG00000204767.4 | INSYN2B | -3.14 | 1.54E-05 | 6.32E-04 |
| ENSG00000180616.9 | SSTR2 | -2.02 | 1.58E-05 | 6.46E-04 |
| ENSG00000188060.7 | RAB42 | -1.54 | 1.98E-05 | 7.71E-04 |
| ENSG00000161798.7 | AQP5 | -2.80 | 2.40E-05 | 8.79E-04 |
| ENSG00000016391.11 | CHDH | -2.12 | 2.66E-05 | 9.47E-04 |
| ENSG00000143546.10 | S100A8 | -1.67 | 3.31E-05 | 1.12E-03 |
| ENSG00000125735.10 | TNFSF14 | -2.07 | 4.19E-05 | 1.35E-03 |
| ENSG00000142748.12 | FCN3 | -1.62 | 4.47E-05 | 1.42E-03 |
| ENSG00000156427.8 | FGF18 | -1.60 | 4.83E-05 | 1.51E-03 |
| ENSG00000188257.11 | PLA2G2A | -3.29 | 5.41E-05 | 1.63E-03 |
| ENSG00000196932.12 | TMEM26 | -2.10 | 5.53E-05 | 1.64E-03 |
| ENSG00000169245.6 | CXCL10 | -2.68 | 6.29E-05 | 1.82E-03 |
| ENSG00000087237.12 | CETP | -2.47 | 6.29E-05 | 1.82E-03 |
| ENSG00000172724.12 | CCL19 | -3.45 | 6.77E-05 | 1.92E-03 |
| ENSG00000168824.14 | NSG1 | -2.02 | 8.20E-05 | 2.21E-03 |
| ENSG00000064300.9 | NGFR | -1.69 | 8.51E-05 | 2.27E-03 |
| ENSG00000150594.7 | ADRA2A | -1.66 | 9.40E-05 | 2.45E-03 |
| ENSG00000204644.9 | ZFP57 | -6.80 | 1.05E-04 | 2.66E-03 |
| ENSG00000166558.10 | SLC38A8 | -2.74 | 1.08E-04 | 2.71E-03 |
| ENSG00000162873.14 | KLHDC8A | -2.63 | 1.19E-04 | 2.91E-03 |
| ENSG00000054938.15 | CHRDL2 | -2.71 | 1.29E-04 | 3.08E-03 |
| ENSG00000056736.10 | IL17RB | -2.09 | 1.31E-04 | 3.11E-03 |
| ENSG00000170458.14 | CD14 | -2.20 | 1.43E-04 | 3.31E-03 |
| ENSG00000179242.16 | CDH4 | -2.49 | 1.66E-04 | 3.70E-03 |
| ENSG00000187116.14 | LILRA5 | -1.74 | 1.70E-04 | 3.77E-03 |
| ENSG00000118785.14 | SPP1 | -1.85 | 1.85E-04 | 4.01E-03 |
| ENSG00000101144.13 | BMP7 | -2.25 | 1.97E-04 | 4.19E-03 |
| ENSG00000164647.9 | STEAP1 | -2.31 | 2.03E-04 | 4.28E-03 |
| ENSG00000221864.4 | KRTAP12-2 | -3.48 | 2.27E-04 | 4.65E-03 |
| ENSG00000184661.14 | CDCA2 | -1.51 | 2.29E-04 | 4.66E-03 |
| ENSG00000182566.13 | CLEC4G | -3.31 | 2.34E-04 | 4.73E-03 |
| ENSG00000184350.11 | MRGPRE | -1.93 | 2.63E-04 | 5.15E-03 |
| ENSG00000275395.6 | FCGBP | -2.88 | 2.93E-04 | 5.55E-03 |
| ENSG00000183876.9 | ARSI | -1.77 | 3.21E-04 | 5.99E-03 |
| ENSG00000021461.16 | CYP3A43 | -1.88 | 3.30E-04 | 6.10E-03 |
| ENSG00000164488.12 | DACT2 | -1.60 | 4.92E-04 | 8.36E-03 |
| ENSG00000104951.16 | IL4I1 | -2.59 | 4.97E-04 | 8.43E-03 |
| ENSG00000169385.3 | RNASE2 | -3.33 | 6.06E-04 | 9.67E-03 |
| ENSG00000174527.9 | MYO1H | -2.21 | 6.23E-04 | 9.87E-03 |
| ENSG00000273259.3 | AL049839.2 | -2.01 | 7.07E-04 | 1.09E-02 |
| ENSG00000102837.7 | OLFM4 | -2.07 | 7.39E-04 | 1.13E-02 |
| ENSG00000148604.15 | RGR | -2.27 | 1.02E-03 | 1.43E-02 |
| ENSG00000164089.9 | ETNPPL | -1.58 | 1.12E-03 | 1.53E-02 |
| ENSG00000076826.9 | CAMSAP3 | -2.08 | 1.18E-03 | 1.59E-02 |
| ENSG00000184564.11 | SLITRK6 | -1.91 | 1.21E-03 | 1.62E-02 |
| ENSG00000122420.10 | PTGFR | -2.23 | 1.31E-03 | 1.71E-02 |
| ENSG00000133048.13 | CHI3L1 | -3.36 | 1.34E-03 | 1.74E-02 |
| ENSG00000172602.11 | RND1 | -1.93 | 1.52E-03 | 1.91E-02 |
| ENSG00000222047.8 | C10orf55 | -2.13 | 1.86E-03 | 2.22E-02 |
| ENSG00000255501.3 | CARD18 | -2.82 | 2.04E-03 | 2.36E-02 |
| ENSG00000052850.8 | ALX4 | -2.52 | 2.35E-03 | 2.62E-02 |
| ENSG00000137709.10 | POU2F3 | -3.15 | 2.63E-03 | 2.84E-02 |
| ENSG00000163739.5 | CXCL1 | -2.35 | 2.79E-03 | 2.96E-02 |
| ENSG00000181001.2 | OR52N1 | -2.27 | 2.91E-03 | 3.05E-02 |
| ENSG00000119411.11 | BSPRY | -1.53 | 3.01E-03 | 3.12E-02 |
| ENSG00000101222.12 | SPEF1 | -1.73 | 3.33E-03 | 3.32E-02 |
| ENSG00000176204.13 | LRRTM4 | -1.52 | 3.73E-03 | 3.59E-02 |
| ENSG00000141750.7 | STAC2 | -2.25 | 4.20E-03 | 3.92E-02 |
| ENSG00000132972.19 | RNF17 | -4.23 | 4.25E-03 | 3.96E-02 |
| ENSG00000183019.7 | MCEMP1 | -2.42 | 4.66E-03 | 4.22E-02 |
| ENSG00000124875.10 | CXCL6 | -3.04 | 4.77E-03 | 4.28E-02 |
| ENSG00000188716.5 | DUPD1 | -2.64 | 4.83E-03 | 4.31E-02 |
| ENSG00000085552.17 | IGSF9 | -2.13 | 4.88E-03 | 4.34E-02 |
| ENSG00000183654.9 | MARCHF11 | -1.64 | 4.96E-03 | 4.39E-02 |
| ENSG00000007908.16 | SELE | -2.71 | 5.58E-03 | 4.74E-02 |

**^a^**Log2FoldChange, only the genes with LFC≤ -1.5 are presented.

**Supplementary Table 7. Significantly reduced proteins in proteomics analysis**

| \| **Symbol** \| **EntrezID** \| **Gene_name** \| **ID** \| **p value** \| **padj** \| **LFC^a^** \| \| --- \| --- \| --- \| --- \| --- \| --- \| --- \| \| FAM162A \| 26355 \| family with sequence similarity 162 member A \| Q96A26 \| 1.06E-08 \| 3.11E-03 \| -4.36 \| \| NDUFAB1 \| 4706 \| NADH:ubiquinone oxidoreductase subunit AB1 \| O14561 \| 1.12E-07 \| 1.87E-02 \| -3.86 \| \| GJA1 \| 2697 \| gap junction protein alpha 1 \| P17302 \| 2.77E-07 \| 3.23E-02 \| -3.84 \| \| MATR3 \| 9782 \| matrin 3 \| P43243 \| 3.28E-09 \| 9.95E-04 \| -3.62 \| \| DDX1 \| 1653 \| DEAD-box helicase 1 \| Q92499 \| 4.56E-09 \| 1.38E-03 \| -3.62 \| \| PCMT1 \| 5110 \| protein-L-isoaspartate (D-aspartate) O-methyltransferase \| P22061 \| 3.07E-07 \| 3.40E-02 \| -3.6 \| \| COA4 \| 51287 \| cytochrome c oxidase assembly factor 4 homolog \| Q9NYJ1 \| 3.96E-09 \| 1.21E-03 \| -3.59 \| \| MYH16 \| 84176 \| myosin heavy chain 16 pseudogene \| Q9H6N6 \| 3.95E-09 \| 1.21E-03 \| -3.52 \| \| MFGE8 \| 4240 \| milk fat globule EGF and factor V/VIII domain containing \| Q08431 \| 6.67E-09 \| 2.05E-03 \| -3.52 \| \| FBLN2 \| 2199 \| fibulin 2 \| P98095 \| 1.64E-09 \| 4.30E-04 \| -3.5 \| \| MYH10 \| 4628 \| myosin heavy chain 10 \| P35580 \| 1.03E-09 \| 2.67E-04 \| -3.43 \| \| RPS7 \| 6201 \| ribosomal protein S7 \| P62081 \| 5.15E-08 \| 1.05E-02 \| -3.42 \| \| ACTG1 \| 71 \| actin gamma 1 \| P63261 \| 5.92E-09 \| 1.83E-03 \| -3.4 \| \| HDGF \| 3068 \| heparin binding growth factor \| P51858 \| 4.30E-08 \| 9.49E-03 \| -3.4 \| \| CAPN1 \| 823 \| calpain 1 \| P07384 \| 4.99E-08 \| 1.04E-02 \| -3.37 \| \| SNRPN \| 6638 \| small nuclear ribonucleoprotein polypeptide N \| P63162 \| 4.23E-09 \| 1.29E-03 \| -3.34 \| \| TMEM43 \| 79188 \| transmembrane protein 43 \| Q9BTV4 \| 1.29E-08 \| 3.60E-03 \| -3.31 \| \| NAPA \| 8775 \| NSF attachment protein alpha \| P54920 \| 8.15E-10 \| 2.28E-04 \| -3.3 \| \| HNRNPH1 \| 3187 \| heterogeneous nuclear ribonucleoprotein H1 \| P31943 \| 9.53E-10 \| 2.55E-04 \| -3.3 \| \| TOLLIP \| 54472 \| toll interacting protein \| Q9H0E2 \| 8.98E-08 \| 1.61E-02 \| -3.3 \| \| APOA1BP \|  \|  \| Q8NCW5 \| 1.23E-08 \| 3.48E-03 \| -3.29 \| \| GARS \|  \|  \| P41250 \| 2.65E-08 \| 6.72E-03 \| -3.29 \| \| CAPZA1 \| 829 \| capping actin protein of muscle Z-line subunit alpha 1 \| P52907 \| 7.05E-09 \| 2.16E-03 \| -3.28 \| \| CLIC1 \| 1192 \| chloride intracellular channel 1 \| O00299 \| 4.03E-08 \| 9.10E-03 \| -3.28 \| \| BCS1L \| 617 \| BCS1 homolog. ubiquinol-cytochrome c reductase complex chaperone \| Q9Y276 \| 1.60E-09 \| 4.22E-04 \| -3.27 \| \| CTTN \| 2017 \| Cortactin \| Q14247 \| 2.89E-08 \| 7.19E-03 \| -3.27 \| \| PSMD12 \| 5718 \| proteasome 26S subunit. non-ATPase 12 \| O00232 \| 1.01E-07 \| 1.74E-02 \| -3.26 \| \| PLCL1 \| 5334 \| phospholipase C like 1 (inactive) \| Q15111 \| 3.12E-09 \| 9.42E-04 \| -3.24 \| \| ABAT \| 18 \| 4-aminobutyrate aminotransferase \| P80404 \| 1.68E-08 \| 4.52E-03 \| -3.22 \| \| CCT5 \| 22948 \| chaperonin containing TCP1 subunit 5 \| P48643 \| 1.09E-07 \| 1.83E-02 \| -3.22 \| \| COA6 \| 388753 \| cytochrome c oxidase assembly factor 6 \| Q5JTJ3 \| 5.51E-07 \| 4.83E-02 \| -3.22 \| \| HNRNPM \| 4670 \| heterogeneous nuclear ribonucleoprotein M \| P52272 \| 8.04E-10 \| 2.26E-04 \| -3.18 \| \| RPSA \| 3921 \| ribosomal protein SA \| P08865 \| 1.85E-09 \| 4.75E-04 \| -3.18 \| \| FKBP3 \| 2287 \| FKBP prolyl isomerase 3 \| Q00688 \| 2.05E-07 \| 2.75E-02 \| -3.18 \| \| COQ3 \| 51805 \| coenzyme Q3. methyltransferase \| Q9NZJ6 \| 3.13E-07 \| 3.44E-02 \| -3.17 \| \| H2AFY \|  \|  \| O75367 \| 5.86E-07 \| 4.99E-02 \| -3.17 \| \| PSMB3 \| 5691 \| proteasome 20S subunit beta 3 \| P49720 \| 2.38E-10 \| 9.69E-05 \| -3.15 \| \| UBAP2L \| 9898 \| ubiquitin associated protein 2 like \| Q14157 \| 7.06E-10 \| 2.13E-04 \| -3.15 \| \| RPLP1 \| 6176 \| ribosomal protein lateral stalk subunit P1 \| P05386 \| 9.87E-09 \| 2.94E-03 \| -3.15 \| \| ARPC1A \| 10552 \| actin related protein 2/3 complex subunit 1A \| Q92747 \| 9.06E-08 \| 1.62E-02 \| -3.14 \| \| OTUB1 \| 55611 \| OTU deubiquitinase. ubiquitin aldehyde binding 1 \| Q96FW1 \| 3.36E-10 \| 1.07E-04 \| -3.13 \| \| TIMM50 \| 92609 \| translocase of inner mitochondrial membrane 50 \| Q3ZCQ8 \| 1.84E-08 \| 4.86E-03 \| -3.13 \| \| LPL \| 4023 \| lipoprotein lipase \| P06858 \| 2.70E-08 \| 6.81E-03 \| -3.13 \| \| HSD17B4 \| 3295 \| hydroxysteroid 17-beta dehydrogenase 4 \| P51659 \| 1.81E-09 \| 4.66E-04 \| -3.12 \| \| ATP6V1E1 \| 529 \| ATPase H+ transporting V1 subunit E1 \| P36543 \| 1.01E-07 \| 1.75E-02 \| -3.12 \| \| RPN1 \| 6184 \| ribophorin I \| P04843 \| 1.86E-08 \| 4.91E-03 \| -3.11 \| \| PSMD11 \| 5717 \| proteasome 26S subunit. non-ATPase 11 \| O00231 \| 3.97E-08 \| 9.02E-03 \| -3.11 \| \| PSMD6 \| 9861 \| proteasome 26S subunit. non-ATPase 6 \| Q15008 \| 5.32E-08 \| 1.07E-02 \| -3.1 \| \| KPNA4 \| 3840 \| karyopherin subunit alpha 4 \| O00629 \| 2.14E-07 \| 2.81E-02 \| -3.1 \| \| RPN2 \| 6185 \| ribophorin II \| P04844 \| 4.69E-07 \| 4.41E-02 \| -3.1 \| \| SLC2A4 \| 6517 \| solute carrier family 2 member 4 \| P14672 \| 9.04E-09 \| 2.73E-03 \| -3.09 \| \| PSMD1 \| 5707 \| proteasome 26S subunit. non-ATPase 1 \| Q99460 \| 1.35E-08 \| 3.72E-03 \| -3.09 \| \| DNAJA2 \| 10294 \| DnaJ heat shock protein family (Hsp40) member A2 \| O60884 \| 2.12E-07 \| 2.80E-02 \| -3.09 \| \| PMPCB \| 9512 \| peptidase. mitochondrial processing subunit beta \| O75439 \| 2.31E-07 \| 2.94E-02 \| -3.08 \| \| GPX1 \| 2876 \| glutathione peroxidase 1 \| P07203 \| 7.82E-10 \| 2.24E-04 \| -3.07 \| \| DCTN1 \| 1639 \| dynactin subunit 1 \| Q14203 \| 4.91E-08 \| 1.03E-02 \| -3.07 \| \| C8G \| 733 \| complement C8 gamma chain \| P07360 \| 1.46E-07 \| 2.23E-02 \| -3.07 \| \| BANF1 \| 8815 \| BAF nuclear assembly factor 1 \| O75531 \| 2.60E-07 \| 3.13E-02 \| -3.07 \| \| ILF2 \| 3608 \| interleukin enhancer binding factor 2 \| Q12905 \| 2.61E-07 \| 3.14E-02 \| -3.06 \| \| PSMC4 \| 5704 \| proteasome 26S subunit. ATPase 4 \| P43686 \| 4.08E-07 \| 4.06E-02 \| -3.06 \| \| ASNA1 \|  \|  \| O43681 \| 4.12E-08 \| 9.24E-03 \| -3.04 \| \| BCL2L13 \| 23786 \| BCL2 like 13 \| Q9BXK5 \| 5.74E-09 \| 1.78E-03 \| -3.03 \| \| SYNCRIP \| 10492 \| synaptotagmin binding cytoplasmic RNA interacting protein \| O60506 \| 1.17E-08 \| 3.36E-03 \| -3.03 \| \| RPL29 \| 6159 \| ribosomal protein L29 \| P47914 \| 3.97E-08 \| 9.01E-03 \| -3.03 \| \| SPG7 \| 6687 \| SPG7 matrix AAA peptidase subunit. paraplegin \| Q9UQ90 \| 5.09E-08 \| 1.05E-02 \| -3.03 \| \| SGCA \| 6442 \| sarcoglycan alpha \| Q16586 \| 1.05E-07 \| 1.80E-02 \| -3.03 \| \| RPLP0P6 \| 220717 \| ribosomal protein lateral stalk subunit P0 pseudogene 6 \| Q8NHW5 \| 3.86E-08 \| 8.85E-03 \| -3.02 \| \| FIS1 \| 51024 \| fission. mitochondrial 1 \| Q9Y3D6 \| 2.12E-07 \| 2.80E-02 \| -3.02 \| \| CCDC141 \| 285025 \| coiled-coil domain containing 141 \| Q6ZP82 \| 6.33E-08 \| 1.24E-02 \| -3.01 \| \| MLYCD \| 23417 \| malonyl-CoA decarboxylase \| O95822 \| 1.15E-07 \| 1.90E-02 \| -3.01 \| \| UBE2D4 \| 51619 \| ubiquitin conjugating enzyme E2 D4 (putative) \| Q9Y2X8 \| 2.85E-07 \| 3.28E-02 \| -3.01 \| \| MYH2 \| 4620 \| myosin heavy chain 2 \| Q9UKX2 \| 3.10E-07 \| 3.42E-02 \| -3.01 \| \| GLOD4 \| 51031 \| glyoxalase domain containing 4 \| Q9HC38 \| 3.75E-07 \| 3.86E-02 \| -3.01 \| \| MARCKS \| 4082 \| myristoylated alanine rich protein kinase C substrate \| P29966 \| 5.50E-07 \| 4.83E-02 \| -3.01 \| \| CMC1 \| 152100 \| C-X9-C motif containing 1 \| Q7Z7K0 \| 9.38E-08 \| 1.66E-02 \| -3 \| \| TMLHE \| 55217 \| trimethyllysine hydroxylase, epsilon \| Q9NVH6 \| 2.68E-08 \| 6.79E-03 \| -2.98 \| \| NLRX1 \| 79671 \| NLR family member X1 \| Q86UT6 \| 2.96E-08 \| 7.33E-03 \| -2.98 \| \| HLA-A \| 3105 \| major histocompatibility complex, class I, A \| P04439 \| 4.17E-07 \| 4.12E-02 \| -2.97 \| \| HSPB3 \| 8988 \| heat shock protein family B (small) member 3 \| Q12988 \| 5.30E-08 \| 1.07E-02 \| -2.96 \| \| BZW2 \| 28969 \| basic leucine zipper and W2 domains 2 \| Q9Y6E2 \| 1.29E-08 \| 3.60E-03 \| -2.94 \| \| SFPQ \| 6421 \| splicing factor proline and glutamine rich \| P23246 \| 1.21E-09 \| 3.18E-04 \| -2.92 \| \| CLPB \| 81570 \| caseinolytic mitochondrial matrix peptidase chaperone subunit B \| Q9H078 \| 1.47E-07 \| 2.23E-02 \| -2.92 \| \| SUOX \| 6821 \| sulfite oxidase \| P51687 \| 4.06E-08 \| 9.15E-03 \| -2.89 \| \| ATIC \| 471 \| 5-aminoimidazole-4-carboxamide ribonucleotide formyltransferase/IMP cyclohydrolase \| P31939 \| 1.59E-08 \| 4.31E-03 \| -2.88 \| \| GSTM5 \| 2949 \| glutathione S-transferase mu 5 \| P46439 \| 1.39E-07 \| 2.16E-02 \| -2.87 \| \| LIMS1 \| 3987 \| LIM zinc finger domain containing 1 \| P48059 \| 1.60E-07 \| 2.36E-02 \| -2.85 \| \| CAP1 \| 10487 \| cyclase associated actin cytoskeleton regulatory protein 1 \| Q01518 \| 5.81E-07 \| 4.97E-02 \| -2.84 \| \| TOM1L2 \| 146691 \| target of myb1 like 2 membrane trafficking protein \| Q6ZVM7 \| 8.44E-08 \| 1.54E-02 \| -2.82 \| \| DDOST \| 1650 \| dolichyl-diphosphooligosaccharide--protein glycosyltransferase non-catalytic subunit \| P39656 \| 8.50E-09 \| 2.58E-03 \| -2.81 \| \| USP14 \| 9097 \| ubiquitin specific peptidase 14 \| P54578 \| 3.07E-07 \| 3.40E-02 \| -2.81 \| \| SEPT11 \|  \|  \| Q9NVA2 \| 3.14E-07 \| 3.44E-02 \| -2.81 \| \| RPS2 \| 6187 \| ribosomal protein S2 \| P15880 \| 1.51E-07 \| 2.27E-02 \| -2.78 \| \| FLOT1 \| 10211 \| flotillin 1 \| O75955 \| 1.19E-07 \| 1.94E-02 \| -2.76 \| \| TXN2 \| 25828 \| thioredoxin 2 \| Q99757 \| 3.05E-07 \| 3.39E-02 \| -2.75 \| |
| --- | --- | --- | --- | --- | --- | --- | --- | --- | --- | --- | --- | --- | --- | --- | --- | --- | --- | --- | --- | --- | --- | --- | --- | --- | --- | --- | --- | --- | --- | --- | --- | --- | --- | --- | --- | --- | --- | --- | --- | --- | --- | --- | --- | --- | --- | --- | --- | --- | --- | --- | --- | --- | --- | --- | --- | --- | --- | --- | --- | --- | --- | --- | --- | --- | --- | --- | --- | --- | --- | --- | --- | --- | --- | --- | --- | --- | --- | --- | --- | --- | --- | --- | --- | --- | --- | --- | --- | --- | --- | --- | --- | --- | --- | --- | --- | --- | --- | --- | --- | --- | --- | --- | --- | --- | --- | --- | --- | --- | --- | --- | --- | --- | --- | --- | --- | --- | --- | --- | --- | --- | --- | --- | --- | --- | --- | --- | --- | --- | --- | --- | --- | --- | --- | --- | --- | --- | --- | --- | --- | --- | --- | --- | --- | --- | --- | --- | --- | --- | --- | --- | --- | --- | --- | --- | --- | --- | --- | --- | --- | --- | --- | --- | --- | --- | --- | --- | --- | --- | --- | --- | --- | --- | --- | --- | --- | --- | --- | --- | --- | --- | --- | --- | --- | --- | --- | --- | --- | --- | --- | --- | --- | --- | --- | --- | --- | --- | --- | --- | --- | --- | --- | --- | --- | --- | --- | --- | --- | --- | --- | --- | --- | --- | --- | --- | --- | --- | --- | --- | --- | --- | --- | --- | --- | --- | --- | --- | --- | --- | --- | --- | --- | --- | --- | --- | --- | --- | --- | --- | --- | --- | --- | --- | --- | --- | --- | --- | --- | --- | --- | --- | --- | --- | --- | --- | --- | --- | --- | --- | --- | --- | --- | --- | --- | --- | --- | --- | --- | --- | --- | --- | --- | --- | --- | --- | --- | --- | --- | --- | --- | --- | --- | --- | --- | --- | --- | --- | --- | --- | --- | --- | --- | --- | --- | --- | --- | --- | --- | --- | --- | --- | --- | --- | --- | --- | --- | --- | --- | --- | --- | --- | --- | --- | --- | --- | --- | --- | --- | --- | --- | --- | --- | --- | --- | --- | --- | --- | --- | --- | --- | --- | --- | --- | --- | --- | --- | --- | --- | --- | --- | --- | --- | --- | --- | --- | --- | --- | --- | --- | --- | --- | --- | --- | --- | --- | --- | --- | --- | --- | --- | --- | --- | --- | --- | --- | --- | --- | --- | --- | --- | --- | --- | --- | --- | --- | --- | --- | --- | --- | --- | --- | --- | --- | --- | --- | --- | --- | --- | --- | --- | --- | --- | --- | --- | --- | --- | --- | --- | --- | --- | --- | --- | --- | --- | --- | --- | --- | --- | --- | --- | --- | --- | --- | --- | --- | --- | --- | --- | --- | --- | --- | --- | --- | --- | --- | --- | --- | --- | --- | --- | --- | --- | --- | --- | --- | --- | --- | --- | --- | --- | --- | --- | --- | --- | --- | --- | --- | --- | --- | --- | --- | --- | --- | --- | --- | --- | --- | --- | --- | --- | --- | --- | --- | --- | --- | --- | --- | --- | --- | --- | --- | --- | --- | --- | --- | --- | --- | --- | --- | --- | --- | --- | --- | --- | --- | --- | --- | --- | --- | --- | --- | --- | --- | --- | --- | --- | --- | --- | --- | --- | --- | --- | --- | --- | --- | --- | --- | --- | --- | --- | --- | --- | --- | --- | --- | --- | --- | --- | --- | --- | --- | --- | --- | --- | --- | --- | --- | --- | --- | --- | --- | --- | --- | --- | --- | --- | --- | --- | --- | --- | --- | --- | --- | --- | --- | --- | --- | --- | --- | --- | --- | --- | --- | --- | --- | --- | --- | --- | --- | --- | --- | --- | --- | --- | --- | --- | --- | --- | --- | --- | --- | --- | --- | --- | --- | --- | --- | --- | --- | --- | --- | --- | --- | --- | --- | --- | --- | --- | --- | --- | --- | --- | --- | --- | --- | --- | --- | --- | --- | --- | --- | --- | --- | --- | --- | --- | --- | --- | --- | --- | --- | --- | --- | --- | --- | --- | --- | --- | --- | --- | --- | --- | --- | --- | --- | --- | --- | --- | --- | --- | --- | --- | --- | --- | --- | --- | --- | --- | --- | --- | --- | --- | --- | --- | --- | --- | --- | --- | --- | --- | --- | --- | --- | --- | --- | --- | --- | --- | --- | --- | --- | --- | --- | --- | --- | --- |

**^a^**Log2FoldChange

**Supplementary Table 8. Top 50 signaling pathways in integrated Reactome enrichment**

| **ID** | **Description** | **GeneRatio** | **BgRatio** | **p value** | **padj** | **q value** |
| --- | --- | --- | --- | --- | --- | --- |
| R-HSA-9006925 | Intracellular signaling by second messengers | 64/1100 | 306/10704 | 1.81E-08 | 2.40E-05 | 2.04E-05 |
| R-HSA-449147 | Signaling by Interleukins | 85/1100 | 461/10704 | 4.38E-08 | 2.40E-05 | 2.04E-05 |
| R-HSA-9607240 | FLT3 Signaling | 61/1100 | 294/10704 | 5.35E-08 | 2.40E-05 | 2.04E-05 |
| R-HSA-5683057 | MAPK family signaling cascades | 64/1100 | 325/10704 | 1.92E-07 | 6.46E-05 | 5.48E-05 |
| R-HSA-5684996 | MAPK1/MAPK3 signaling | 58/1100 | 286/10704 | 2.59E-07 | 6.46E-05 | 5.48E-05 |
| R-HSA-5673001 | RAF/MAP kinase cascade | 57/1100 | 280/10704 | 2.88E-07 | 6.46E-05 | 5.48E-05 |
| R-HSA-5663202 | Diseases of signal transduction by growth factor receptors and second messengers | 70/1100 | 387/10704 | 1.47E-06 | 2.82E-04 | 2.39E-04 |
| R-HSA-9008059 | Interleukin-37 signaling | 11/1100 | 21/10704 | 1.71E-06 | 2.88E-04 | 2.45E-04 |
| R-HSA-2219528 | PI3K/AKT Signaling in Cancer | 27/1100 | 101/10704 | 2.22E-06 | 3.32E-04 | 2.81E-04 |
| R-HSA-446652 | Interleukin-1 family signaling | 33/1100 | 140/10704 | 3.69E-06 | 4.97E-04 | 4.21E-04 |
| R-HSA-1257604 | PIP3 activates AKT signaling | 51/1100 | 264/10704 | 6.00E-06 | 7.34E-04 | 6.22E-04 |
| R-HSA-194840 | Rho GTPase cycle | 32/1100 | 138/10704 | 7.45E-06 | 8.36E-04 | 7.09E-04 |
| R-HSA-8878166 | Transcriptional regulation by RUNX2 | 29/1100 | 121/10704 | 1.00E-05 | 1.04E-03 | 8.81E-04 |
| R-HSA-194315 | Signaling by Rho GTPases | 75/1100 | 455/10704 | 2.06E-05 | 1.98E-03 | 1.68E-03 |
| R-HSA-111447 | Activation of BAD and translocation to mitochondria | 8/1100 | 15/10704 | 4.03E-05 | 3.54E-03 | 3.01E-03 |
| R-HSA-111933 | Calmodulin induced events | 13/1100 | 37/10704 | 4.47E-05 | 3.54E-03 | 3.01E-03 |
| R-HSA-111997 | CaM pathway | 13/1100 | 37/10704 | 4.47E-05 | 3.54E-03 | 3.01E-03 |
| R-HSA-1489509 | DAG and IP3 signaling | 14/1100 | 43/10704 | 6.04E-05 | 4.35E-03 | 3.69E-03 |
| R-HSA-9634638 | Estrogen-dependent nuclear events downstream of ESR-membrane signaling | 10/1100 | 24/10704 | 6.41E-05 | 4.35E-03 | 3.69E-03 |
| R-HSA-9614085 | FOXO-mediated transcription | 18/1100 | 65/10704 | 6.46E-05 | 4.35E-03 | 3.69E-03 |
| R-HSA-111996 | Ca-dependent events | 13/1100 | 39/10704 | 8.39E-05 | 5.38E-03 | 4.56E-03 |
| R-HSA-6811558 | PI5P, PP2A and IER3 Regulate PI3K/AKT Signaling | 24/1100 | 103/10704 | 9.23E-05 | 5.65E-03 | 4.79E-03 |
| R-HSA-199418 | Negative regulation of the PI3K/AKT network | 25/1100 | 110/10704 | 1.02E-04 | 5.99E-03 | 5.08E-03 |
| R-HSA-111931 | PKA-mediated phosphorylation of CREB | 9/1100 | 22/10704 | 1.78E-04 | 9.98E-03 | 8.47E-03 |
| R-HSA-194138 | Signaling by VEGF | 24/1100 | 108/10704 | 2.02E-04 | 1.09E-02 | 9.22E-03 |
| R-HSA-9614399 | Regulation of localization of FOXO transcription factors | 6/1100 | 11/10704 | 3.40E-04 | 1.75E-02 | 1.48E-02 |
| R-HSA-2029480 | Fcgamma receptor (FCGR) dependent phagocytosis | 20/1100 | 86/10704 | 3.56E-04 | 1.75E-02 | 1.48E-02 |
| R-HSA-2032785 | YAP1- and WWTR1 (TAZ)-stimulated gene expression | 7/1100 | 15/10704 | 3.63E-04 | 1.75E-02 | 1.48E-02 |
| R-HSA-2219530 | Constitutive Signaling by Aberrant PI3K in Cancer | 18/1100 | 75/10704 | 4.60E-04 | 2.14E-02 | 1.81E-02 |
| R-HSA-114604 | GPVI-mediated activation cascade | 11/1100 | 35/10704 | 5.23E-04 | 2.32E-02 | 1.97E-02 |
| R-HSA-5674400 | Constitutive Signaling by AKT1 E17K in Cancer | 9/1100 | 25/10704 | 5.50E-04 | 2.32E-02 | 1.97E-02 |
| R-HSA-4090294 | SUMOylation of intracellular receptors | 10/1100 | 30/10704 | 5.53E-04 | 2.32E-02 | 1.97E-02 |
| R-HSA-9658195 | Leishmania infection | 43/1100 | 252/10704 | 5.68E-04 | 2.32E-02 | 1.97E-02 |
| R-HSA-6804757 | Regulation of TP53 Degradation | 11/1100 | 36/10704 | 6.84E-04 | 2.71E-02 | 2.30E-02 |
| R-HSA-512988 | Interleukin-3. Interleukin-5 and GM-CSF signaling | 13/1100 | 48/10704 | 8.29E-04 | 3.00E-02 | 2.54E-02 |
| R-HSA-3108232 | SUMO E3 ligases SUMOylate target proteins | 33/1100 | 182/10704 | 8.30E-04 | 3.00E-02 | 2.54E-02 |
| R-HSA-74751 | Insulin receptor signalling cascade | 14/1100 | 54/10704 | 8.52E-04 | 3.00E-02 | 2.54E-02 |
| R-HSA-6806003 | Regulation of TP53 Expression and Degradation | 11/1100 | 37/10704 | 8.84E-04 | 3.00E-02 | 2.54E-02 |
| R-HSA-5357801 | Programmed Cell Death | 34/1100 | 190/10704 | 8.88E-04 | 3.00E-02 | 2.54E-02 |
| R-HSA-6806834 | Signaling by MET | 18/1100 | 79/10704 | 8.92E-04 | 3.00E-02 | 2.54E-02 |
| R-HSA-164378 | PKA activation in glucagon signalling | 7/1100 | 17/10704 | 9.13E-04 | 3.00E-02 | 2.54E-02 |
| R-HSA-8862803 | Deregulated CDK5 triggers multiple neurodegenerative pathways in Alzheimer's disease models | 8/1100 | 22/10704 | 1.04E-03 | 3.25E-02 | 2.76E-02 |
| R-HSA-8863678 | Neurodegenerative Diseases | 8/1100 | 22/10704 | 1.04E-03 | 3.25E-02 | 2.76E-02 |
| R-HSA-109581 | Apoptosis | 32/1100 | 178/10704 | 1.15E-03 | 3.51E-02 | 2.97E-02 |
| R-HSA-109704 | PI3K Cascade | 12/1100 | 44/10704 | 1.22E-03 | 3.57E-02 | 3.03E-02 |
| R-HSA-451927 | Interleukin-2 family signaling | 12/1100 | 44/10704 | 1.22E-03 | 3.57E-02 | 3.03E-02 |
| R-HSA-76002 | Platelet activation. signaling and aggregation | 43/1100 | 263/10704 | 1.38E-03 | 3.96E-02 | 3.36E-02 |
| R-HSA-2990846 | SUMOylation | 33/1100 | 188/10704 | 1.47E-03 | 4.12E-02 | 3.49E-02 |
| R-HSA-69275 | G2/M Transition | 34/1100 | 196/10704 | 1.54E-03 | 4.19E-02 | 3.56E-02 |
| R-HSA-8878159 | Transcriptional regulation by RUNX3 | 20/1100 | 96/10704 | 1.56E-03 | 4.19E-02 | 3.56E-02 |

**Supplementary Table 9. Top 50 signaling pathways in integrated KEGG enrichment**

| **ID** | **Description** | **GeneRatio** | **BgRatio** | **p value** | **padj** | **q value** |
| --- | --- | --- | --- | --- | --- | --- |
| hsa04151 | PI3K-Akt signaling pathway | 78/776 | 354/8102 | 6.54E-13 | 2.05E-10 | 1.27E-10 |
| hsa04910 | Insulin signaling pathway | 42/776 | 137/8102 | 2.83E-12 | 4.44E-10 | 2.76E-10 |
| hsa04010 | MAPK signaling pathway | 67/776 | 294/8102 | 6.20E-12 | 6.47E-10 | 4.02E-10 |
| hsa04014 | Ras signaling pathway | 51/776 | 232/8102 | 8.53E-09 | 6.68E-07 | 4.15E-07 |
| hsa01521 | EGFR tyrosine kinase inhibitor resistance | 25/776 | 79/8102 | 3.82E-08 | 2.39E-06 | 1.49E-06 |
| hsa04510 | Focal adhesion | 44/776 | 201/8102 | 1.05E-07 | 5.47E-06 | 3.40E-06 |
| hsa04015 | Rap1 signaling pathway | 45/776 | 210/8102 | 1.45E-07 | 6.50E-06 | 4.04E-06 |
| hsa05215 | Prostate cancer | 27/776 | 97/8102 | 2.17E-07 | 8.49E-06 | 5.28E-06 |
| hsa04022 | cGMP-PKG signaling pathway | 38/776 | 167/8102 | 2.77E-07 | 9.62E-06 | 5.98E-06 |
| hsa04722 | Neurotrophin signaling pathway | 30/776 | 119/8102 | 5.00E-07 | 1.57E-05 | 9.74E-06 |
| hsa04931 | Insulin resistance | 28/776 | 108/8102 | 6.49E-07 | 1.85E-05 | 1.15E-05 |
| hsa04218 | Cellular senescence | 35/776 | 156/8102 | 1.16E-06 | 3.04E-05 | 1.89E-05 |
| hsa05212 | Pancreatic cancer | 22/776 | 76/8102 | 1.39E-06 | 3.36E-05 | 2.09E-05 |
| hsa05223 | Non-small cell lung cancer | 21/776 | 72/8102 | 2.11E-06 | 4.72E-05 | 2.94E-05 |
| hsa01522 | Endocrine resistance | 25/776 | 98/8102 | 3.54E-06 | 7.38E-05 | 4.59E-05 |
| hsa05220 | Chronic myeloid leukemia | 21/776 | 76/8102 | 5.48E-06 | 1.02E-04 | 6.34E-05 |
| hsa04024 | cAMP signaling pathway | 42/776 | 216/8102 | 5.54E-06 | 1.02E-04 | 6.34E-05 |
| hsa04210 | Apoptosis | 30/776 | 136/8102 | 9.65E-06 | 1.58E-04 | 9.86E-05 |
| hsa04666 | Fc gamma R-mediated phagocytosis | 24/776 | 97/8102 | 9.76E-06 | 1.58E-04 | 9.86E-05 |
| hsa05221 | Acute myeloid leukemia | 19/776 | 67/8102 | 1.01E-05 | 1.58E-04 | 9.86E-05 |
| hsa05169 | Epstein-Barr virus infection | 39/776 | 202/8102 | 1.42E-05 | 2.12E-04 | 1.32E-04 |
| hsa05166 | Human T-cell leukemia virus 1 infection | 41/776 | 219/8102 | 1.86E-05 | 2.65E-04 | 1.65E-04 |
| hsa04211 | Longevity regulating pathway | 22/776 | 89/8102 | 2.31E-05 | 3.15E-04 | 1.96E-04 |
| hsa04064 | NF-kappa B signaling pathway | 24/776 | 104/8102 | 3.41E-05 | 4.29E-04 | 2.67E-04 |
| hsa04068 | FoxO signaling pathway | 28/776 | 131/8102 | 3.51E-05 | 4.29E-04 | 2.67E-04 |
| hsa04012 | ErbB signaling pathway | 21/776 | 85/8102 | 3.56E-05 | 4.29E-04 | 2.67E-04 |
| hsa04935 | Growth hormone synthesis, secretion and action | 26/776 | 119/8102 | 4.46E-05 | 5.17E-04 | 3.22E-04 |
| hsa04933 | AGE-RAGE signaling pathway in diabetic complications | 23/776 | 100/8102 | 5.23E-05 | 5.84E-04 | 3.63E-04 |
| hsa04062 | Chemokine signaling pathway | 36/776 | 192/8102 | 5.78E-05 | 6.24E-04 | 3.88E-04 |
| hsa04625 | C-type lectin receptor signaling pathway | 23/776 | 104/8102 | 9.97E-05 | 1.04E-03 | 6.47E-04 |
| hsa05418 | Fluid shear stress and atherosclerosis | 28/776 | 139/8102 | 1.06E-04 | 1.07E-03 | 6.67E-04 |
| hsa04270 | Vascular smooth muscle contraction | 27/776 | 133/8102 | 1.22E-04 | 1.19E-03 | 7.38E-04 |
| hsa05161 | Hepatitis B | 31/776 | 162/8102 | 1.26E-04 | 1.19E-03 | 7.38E-04 |
| hsa04072 | Phospholipase D signaling pathway | 29/776 | 148/8102 | 1.34E-04 | 1.19E-03 | 7.38E-04 |
| hsa04928 | Parathyroid hormone synthesis, secretion and action | 23/776 | 106/8102 | 1.35E-04 | 1.19E-03 | 7.38E-04 |
| hsa05131 | Shigellosis | 42/776 | 246/8102 | 1.36E-04 | 1.19E-03 | 7.38E-04 |
| hsa04810 | Regulation of actin cytoskeleton | 38/776 | 218/8102 | 1.82E-04 | 1.54E-03 | 9.57E-04 |
| hsa04371 | Apelin signaling pathway | 27/776 | 137/8102 | 2.05E-04 | 1.69E-03 | 1.05E-03 |
| hsa04066 | HIF-1 signaling pathway | 23/776 | 109/8102 | 2.11E-04 | 1.69E-03 | 1.05E-03 |
| hsa05205 | Proteoglycans in cancer | 36/776 | 205/8102 | 2.30E-04 | 1.80E-03 | 1.12E-03 |
| hsa04611 | Platelet activation | 25/776 | 124/8102 | 2.42E-04 | 1.85E-03 | 1.15E-03 |
| hsa05225 | Hepatocellular carcinoma | 31/776 | 168/8102 | 2.51E-04 | 1.87E-03 | 1.16E-03 |
| hsa05213 | Endometrial cancer | 15/776 | 58/8102 | 2.67E-04 | 1.94E-03 | 1.21E-03 |
| hsa04630 | JAK-STAT signaling pathway | 30/776 | 162/8102 | 2.94E-04 | 2.09E-03 | 1.30E-03 |
| hsa05231 | Choline metabolism in cancer | 21/776 | 98/8102 | 3.14E-04 | 2.13E-03 | 1.33E-03 |
| hsa04071 | Sphingolipid signaling pathway | 24/776 | 119/8102 | 3.18E-04 | 2.13E-03 | 1.33E-03 |
| hsa04668 | TNF signaling pathway | 23/776 | 112/8102 | 3.20E-04 | 2.13E-03 | 1.33E-03 |
| hsa04020 | Calcium signaling pathway | 40/776 | 240/8102 | 3.29E-04 | 2.15E-03 | 1.34E-03 |
| hsa05210 | Colorectal cancer | 19/776 | 86/8102 | 3.98E-04 | 2.54E-03 | 1.58E-03 |
| hsa01524 | Platinum drug resistance | 17/776 | 73/8102 | 4.15E-04 | 2.55E-03 | 1.58E-03 |

**Supplementary Table 10. Top 50 signaling pathways in integrated GO-BP enrichment**

| **ID** | **Description** | **GeneRatio** | **BgRatio** | **p value** | **padj** | **q value** |
| --- | --- | --- | --- | --- | --- | --- |
| GO:0007265 | Ras protein signal transduction | 75/1666 | 346/18866 | 1.72E-13 | 1.03E-09 | 7.90E-10 |
| GO:1901653 | cellular response to peptide | 78/1666 | 398/18866 | 1.28E-11 | 3.85E-08 | 2.95E-08 |
| GO:0051056 | regulation of small GTPase mediated signal transduction | 67/1666 | 323/18866 | 2.73E-11 | 5.46E-08 | 4.18E-08 |
| GO:0043434 | response to peptide hormone | 82/1666 | 447/18866 | 1.24E-10 | 1.86E-07 | 1.43E-07 |
| GO:0060562 | epithelial tube morphogenesis | 65/1666 | 331/18866 | 6.05E-10 | 7.26E-07 | 5.56E-07 |
| GO:0007266 | Rho protein signal transduction | 37/1666 | 140/18866 | 8.05E-10 | 8.04E-07 | 6.16E-07 |
| GO:0070482 | response to oxygen levels | 73/1666 | 396/18866 | 1.04E-09 | 8.92E-07 | 6.84E-07 |
| GO:0032970 | regulation of actin filament-based process | 74/1666 | 405/18866 | 1.20E-09 | 9.02E-07 | 6.92E-07 |
| GO:0001655 | urogenital system development | 64/1666 | 330/18866 | 1.39E-09 | 9.25E-07 | 7.09E-07 |
| GO:0036293 | response to decreased oxygen levels | 69/1666 | 371/18866 | 2.03E-09 | 1.22E-06 | 9.35E-07 |
| GO:0018108 | peptidyl-tyrosine phosphorylation | 69/1666 | 374/18866 | 2.89E-09 | 1.57E-06 | 1.21E-06 |
| GO:0071375 | cellular response to peptide hormone stimulus | 63/1666 | 330/18866 | 3.56E-09 | 1.78E-06 | 1.36E-06 |
| GO:0018212 | peptidyl-tyrosine modification | 69/1666 | 377/18866 | 4.07E-09 | 1.88E-06 | 1.44E-06 |
| GO:0022604 | regulation of cell morphogenesis | 84/1666 | 499/18866 | 5.50E-09 | 2.35E-06 | 1.80E-06 |
| GO:0007015 | actin filament organization | 75/1666 | 434/18866 | 1.16E-08 | 4.64E-06 | 3.56E-06 |
| GO:0046777 | protein autophosphorylation | 49/1666 | 237/18866 | 1.40E-08 | 5.23E-06 | 4.01E-06 |
| GO:0008360 | regulation of cell shape | 37/1666 | 156/18866 | 1.95E-08 | 6.88E-06 | 5.27E-06 |
| GO:0042391 | regulation of membrane potential | 75/1666 | 443/18866 | 2.84E-08 | 9.45E-06 | 7.25E-06 |
| GO:0046578 | regulation of Ras protein signal transduction | 42/1666 | 194/18866 | 3.78E-08 | 1.18E-05 | 9.03E-06 |
| GO:0001666 | response to hypoxia | 64/1666 | 359/18866 | 4.17E-08 | 1.18E-05 | 9.03E-06 |
| GO:0001659 | temperature homeostasis | 39/1666 | 174/18866 | 4.28E-08 | 1.18E-05 | 9.03E-06 |
| GO:1905475 | regulation of protein localization to membrane | 42/1666 | 195/18866 | 4.41E-08 | 1.18E-05 | 9.03E-06 |
| GO:0019932 | second-messenger-mediated signaling | 76/1666 | 456/18866 | 4.52E-08 | 1.18E-05 | 9.03E-06 |
| GO:0048010 | vascular endothelial growth factor receptor signaling pathway | 27/1666 | 98/18866 | 5.92E-08 | 1.48E-05 | 1.13E-05 |
| GO:0050730 | regulation of peptidyl-tyrosine phosphorylation | 51/1666 | 263/18866 | 6.44E-08 | 1.54E-05 | 1.18E-05 |
| GO:0035265 | organ growth | 41/1666 | 191/18866 | 7.03E-08 | 1.62E-05 | 1.24E-05 |
| GO:0034765 | regulation of ion transmembrane transport | 79/1666 | 489/18866 | 9.60E-08 | 2.13E-05 | 1.63E-05 |
| GO:0032956 | regulation of actin cytoskeleton organization | 63/1666 | 360/18866 | 1.07E-07 | 2.29E-05 | 1.75E-05 |
| GO:0060537 | muscle tissue development | 69/1666 | 409/18866 | 1.17E-07 | 2.35E-05 | 1.80E-05 |
| GO:0001822 | kidney development | 53/1666 | 283/18866 | 1.18E-07 | 2.35E-05 | 1.80E-05 |
| GO:0048568 | embryonic organ development | 74/1666 | 451/18866 | 1.30E-07 | 2.51E-05 | 1.92E-05 |
| GO:0045785 | positive regulation of cell adhesion | 71/1666 | 428/18866 | 1.51E-07 | 2.82E-05 | 2.17E-05 |
| GO:0014706 | striated muscle tissue development | 66/1666 | 389/18866 | 1.77E-07 | 3.21E-05 | 2.46E-05 |
| GO:0043405 | regulation of MAP kinase activity | 60/1666 | 342/18866 | 1.96E-07 | 3.45E-05 | 2.64E-05 |
| GO:0043254 | regulation of protein-containing complex assembly | 73/1666 | 449/18866 | 2.28E-07 | 3.90E-05 | 2.99E-05 |
| GO:0001933 | negative regulation of protein phosphorylation | 72/1666 | 444/18866 | 3.05E-07 | 5.08E-05 | 3.90E-05 |
| GO:0006469 | negative regulation of protein kinase activity | 47/1666 | 246/18866 | 3.30E-07 | 5.25E-05 | 4.02E-05 |
| GO:0072001 | renal system development | 53/1666 | 292/18866 | 3.33E-07 | 5.25E-05 | 4.02E-05 |
| GO:0007517 | muscle organ development | 67/1666 | 407/18866 | 4.48E-07 | 6.89E-05 | 5.28E-05 |
| GO:0008625 | extrinsic apoptotic signaling pathway via death domain receptors | 24/1666 | 89/18866 | 4.90E-07 | 7.34E-05 | 5.63E-05 |
| GO:0016311 | dephosphorylation | 77/1666 | 492/18866 | 5.14E-07 | 7.52E-05 | 5.76E-05 |
| GO:0032868 | response to insulin | 51/1666 | 283/18866 | 6.90E-07 | 9.55E-05 | 7.32E-05 |
| GO:1901654 | response to ketone | 40/1666 | 200/18866 | 7.21E-07 | 9.55E-05 | 7.32E-05 |
| GO:1901655 | cellular response to ketone | 25/1666 | 97/18866 | 7.24E-07 | 9.55E-05 | 7.32E-05 |
| GO:0033673 | negative regulation of kinase activity | 49/1666 | 268/18866 | 7.32E-07 | 9.55E-05 | 7.32E-05 |
| GO:0006470 | protein dephosphorylation | 56/1666 | 323/18866 | 7.33E-07 | 9.55E-05 | 7.32E-05 |
| GO:2001233 | regulation of apoptotic signaling pathway | 67/1666 | 413/18866 | 7.70E-07 | 9.82E-05 | 7.53E-05 |
| GO:0106106 | cold-induced thermogenesis | 32/1666 | 144/18866 | 8.33E-07 | 1.01E-04 | 7.74E-05 |
| GO:0120161 | regulation of cold-induced thermogenesis | 32/1666 | 144/18866 | 8.33E-07 | 1.01E-04 | 7.74E-05 |
| GO:0022407 | regulation of cell-cell adhesion | 70/1666 | 439/18866 | 8.42E-07 | 1.01E-04 | 7.74E-05 |


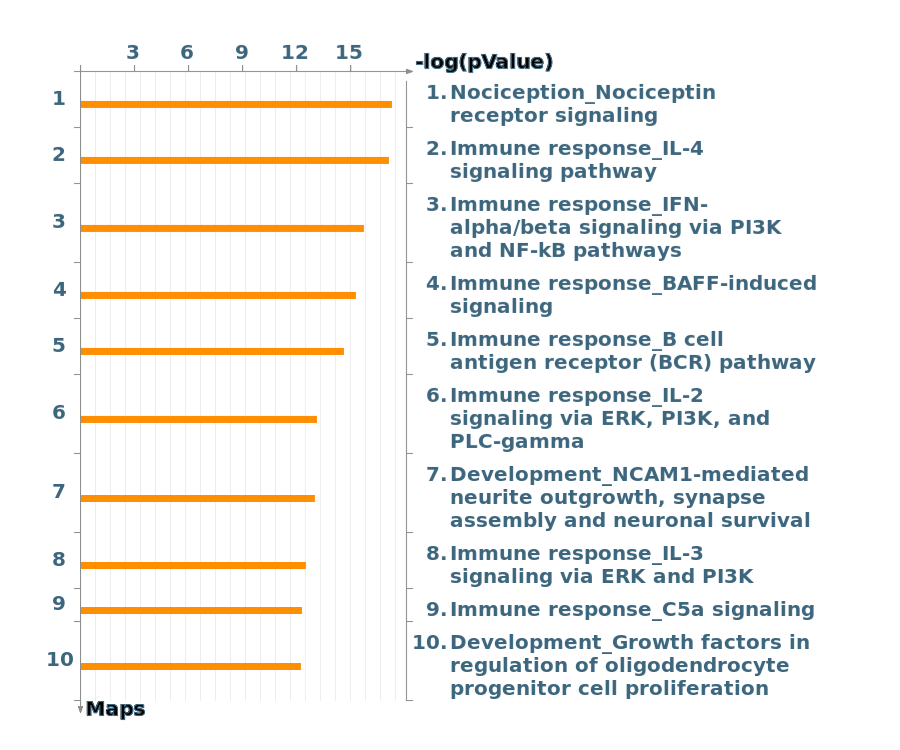


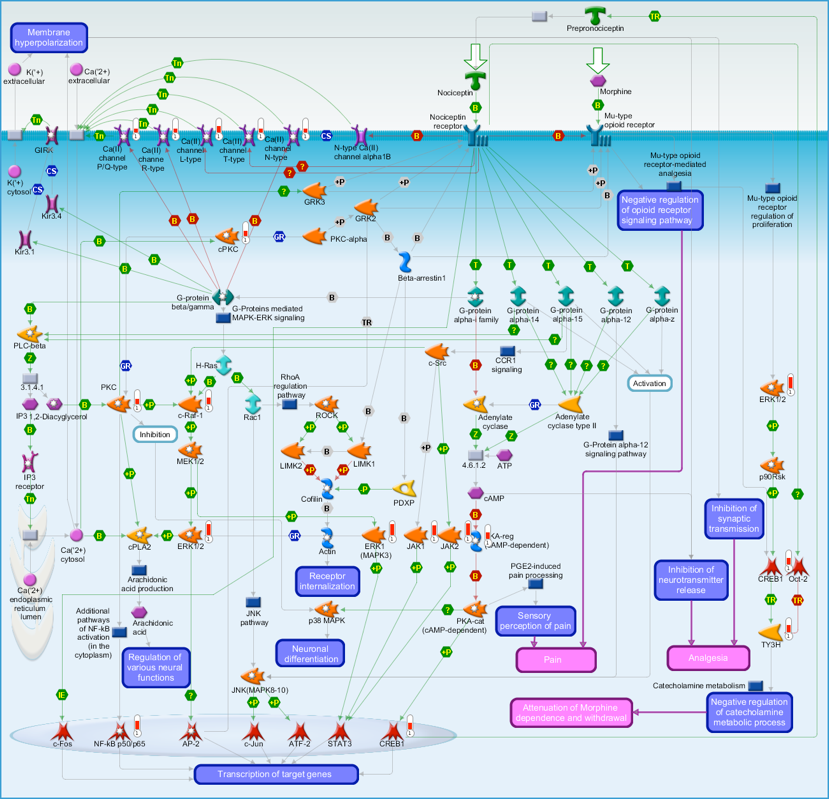


**Supplementary Figure 1. Differential pathway and network interpretation in PamChip kinase activity profiling. A:** Possible canonical pathways responsible for differences in the peptide phosphorylation by Clarivate Analytics® GeneGO Pathway analysis Tool. All maps were created by a high-quality manual curation process based on published peer-reviewed literature. The lower p-value means higher relevance of the entity to the dataset, which shows in higher rating for the entity. **B:** The top scored map with the lowest p-value based on the enrichment distribution and sorted by 'statistically significant maps' set. Experimental data from all files were linked to and visualized on the maps as thermometer-like figures. Up-ward thermometers (red) indicate up-regulated signals and down-ward (blue) ones indicate down-regulated expression levels of the genes.
